# Pan-cancer and single-cell analyses identify CD44 as an immunotherapy response predictor and regulating macrophage polarization and tumor progression in colorectal cancer

**DOI:** 10.3389/fonc.2024.1380821

**Published:** 2024-03-25

**Authors:** Qian Zhang, Xinyu Wang, Yang Liu, Hao Xu, Chun Ye

**Affiliations:** ^1^College of Medicine and Biological Information Engineering, Northeastern University, Shenyang, Liaoning, China; ^2^Department of General Surgery, General Hospital of Northern Theater Command, Shenyang, China; ^3^Department of Pharmacy, Liaoning Cancer Hospital & Institute, Shenyang, Liaoning, China

**Keywords:** CD44, pan cancer, immunotherapy, macrophages, colorectal (colon) cancer

## Abstract

**Introduction:**

Cluster of differentiation (CD) 44 is a non-kinase cell surface transmembrane glycoprotein critical for tumor maintenance and progression.

**Methods:**

We conducted a systematic analysis of the expression profile and genomic alteration profile of CD44 in 33 types of cancer. The immune characteristics of CD44 were comprehensively explored by TIMER2.0 and CIBERSORT. In addition, the CD44 transcriptional landscape was examined at the single-cell level. Then, Pseudotime trajectory analysis of CD44 gene expression was performed using Monocle 2, and CellChat was utilized to compare the crosstalk differences between CD44^+^monocytes and CD44^-^ monocytes. Tumor immune dysfunction and exclusion (TIDE) was used to evaluate the predictive ability of CD44 for immune checkpoint blockade (ICB) responses. The effects of CD44 on colorectal cancer (CRC) and macrophage polarization were investigated by knocking down the expression of CD44 in HCT-116 cell and macrophages *in vitro*.

**Results:**

The expression of CD44 elevated in most cancers, predicting unfavorable prognosis. In addditon, CD44 was correlation with immune cell infiltration and key immune regulators. CD44^+^ monocytes had a higher information flow intensity than CD44- monocytes. CD44 had good predictive ability for immune checkpoint blockade responses. Knockdown of CD44 inhibited the proliferation, migration, and invasion of HCT-116 cell *in vitro*. Knockdown of CD44 inhibited M2 macrophage polarization.

**Discussion:**

These findings suggest that CD44 is involved in regulating tumor development, macrophage polarization, and has certain predictive value for patient clinical prognosis and response to immunotherapy.

## 1 Introduction

Despite the tremendous progress in cancer research, cancer remains the primary threat to global human health (1). ICB therapies, represented by Pembrolizumab and Navulizumab, have shown encouraging therapeutic results in a variety of malignancies, especially in the treatment of non-small cell lung cancer (NSCLC) and melanoma (2, 3). The remarkable clinical success of immunotherapy has ushered in a new era of cancer treatment. However, only a few patients benefit from immune checkpoint inhibitor (ICI) treatment (4, 5). Although microsatellite instability (MSI), tumor mutational burden (TMB), and PD-1/PD-L1 have been recognized as biomarkers predictive of immunotherapy response, their predictive effects are influenced by tumor heterogeneity and individual differences (6–8). Therefore, it is urgent to identify validated predictive biomarkers that more accurately predict the therapeutic effect of ICIs. With the continuous development of sequencing methods, whole exome sequencing (WES) and RNA sequencing (RNA-seq) facilitates genetic spectrum analysis in large populations, while the emergence of single-cell RNA sequncing (scRNA-seq) achieves gene expression profile analysis of each cell at single-cell resolution, which better identifies new biomarkers and provides feasibility for realizing precision medicine (9).

CD44 is a transmembrane glycoprotein with multiple isomers and binds to the extracellular matrix as an adhesion factor to be related to various cellular processes, including cell division, survival, migration, and adhesion (10, 11). The human CD44 gene contains 19 exons, of which 9 variant exons produce multiple CD44 splice variants in different combinations (12). CD44 shows high expression within various cancer cells, which is related to cancer genesis and invasiveness, so it is considered as a molecular marker of cancer stem cells (CSCs) (13–17). Cells with CD44 overexpression exhibit multiple CSC characteristics, including epithelial-mesenchymal transition (EMT), self-renewal, radioresistance and chemoresistance (18, 19). Hyaluronic acid (HA) is a specific CD44 ligand, and CD44 is highly abundant in extracellular matrix (ECM) and can be detected in tumor and stromal cells (20, 21). HA can combine with CD44 ligand binding domain and induces conformational alterations, which is responsible for activating different pathways, causing cell growth, invasion, migration and adhesion (22). Additionally, osteopontin (OPN), called secreted phosphorylated protein 1 (SPP1) as well, represents a sialic acid-rich glycoprotein similar to chemokines, and it acts as the physiological ligand of CD44 on T cells (23) and possible immunotherapeutic target. The role of CD44 in tumor immunomodulation cannot be underestimated. The interaction of CD44-SPP1 has been suggested to inhibit CD8^+^ T cell activation and promote tumor immune tolerance and immune escape (24). CD44 also mediates lymphocyte infiltration, macrophage polarization,and inducing mesenchymal stem cells(MSCs)differentiate into cancer-associated fibroblasts (CAFs) (25–28). Moreover, the CD44-SPP1 axis is critical for cell-cell communication and exerts important immunomodulatory effects in the tumor microenvironment (TME). According to previous studies, the CD44-SPP1 axis mediates crosstalk between tumor cells and macrophages in various cancers, such as glioma, hepatocellular carcinoma, and gastric cancer (29–31).

Currently, most studies have indicated that a high CD44 level predicts a poor prognosis for cancer patients, but some studies have reported opposite results. Therefore, it is necessary to conduct a systematic analysis on the role of CD44 in pan-cancer (32–37). We analyzed the gene expression, mutation characteristics, diagnostic value, and prognostic value of CD44 based on bulk RNA-seq data from The Cancer Genome Atlas (TCGA) database. The effect of CD44 expression on the TME was analyzed, including its correlation with lymphocyte infiltration, immune checkpoint genes, and other immune-related molecules. In addition, we revealed the expression and distribution heterogeneity of the CD44 gene in different cancers at the single-cell level, conducted pseudotime trajectory analysis on CD44 gene expression, and characterized the communication of CD44^+^ monocytes and CD44^-^ monocytes with other cells. Furthermore, we evaluated the feasibility of CD44 as a predictive marker for immunotherapy response using publicly available data. In this study, we confirmed the key role of CD44 in promoting tumor proliferation, migration, and invasion of colorectal tumor cells *in vitro*. In addition, the present study demonstrated that CD44 is essential for the maintenance of the M2 macrophage phenotype. These findings elucidate the regulatory effects of CD44 on tumor progression and TME, which may affect the outcomes of tumor immunotherapy.

## 2 Materials and methods

### 2.1 Data extraction and processing

We acquired bulk RNA-seq data from TCGA (https://portal.gdc.cancer.gov) database, including transcriptomic data and clinical information regarding 33 cancer as well as non-carcinoma samples. Additionally,we obtained scRNA-seq data of six types of cancer, containing breast cancer (GSE176078), CRC (GSE166555), renal cell carcinoma (GSE159115), glioma (GSE135045), gastric cancer (GSE167297) and head and neck squamous cell carcinoma (HNSC) (GSE139324) from Gene Expression Omnibus (GEO) database (https://www.ncbi.nlm.nih.gov/geo/), in which three samples were selected from each dataset and integrated for subsequent data analysis.

### 2.2 CD44 expression in cancer and non-carcinoma samples, and different clinical stages

CD44 mRNA levels in major human and tumor samples, as well as its subcellular localization were explored using Human Protein Atlas (HPA) (https://www.proteinatlas.org/) database,through searching CD44 in Tissue module or Subcell module. GeneeDE module of TIMER2.0 (http://timer.cistrome.org/) database was adopted for analyzing CD44 transcription level within different cancer and non-carcinoma samples. Differential CD44 mRNA expression within normal and cancer samples and at different pathological stages, was performed using Single Gene Analysis module of the Gene Expression Profiling Interactive Analysis (GEPIA) (http://gepia.cancer-pku.cn/) database.

### 2.3 Significance of CD44 in predicting prognosis of cancers

Based on median CD44 gene expression level, we classified patients into low and high CD44 expression groups. Besides, survival package of R software was utilized for survival analysis, followed by plotting of Kaplan-Meier survival curve. Furthermore, univariate Cox regression analysis was utilized for assessing whether CD44 was significant for predicting overall survival (OS) and disease-specific survival (DSS) of patients with cancers.

### 2.4 Landscape of CD44 mutation profile in different tissues

We utilized cBioPortal platform (https://www.cbioportal.org/) for analyzing CD44 mutation frequency and characteristics within pan-cancer from TCGA database. The gene alternations and mutation sites of CD44 were viewed with the OncoPrint module. The impact of CD44 gene alternations on survival was evaluated using tumor sample data from TCGA database with the Comparison/Survival module. We acquired the somatic mutations and somatic copy number alterations of tumor samples from cutaneous melanoma based on TCGA database. The patients were allocated to high or low CD44 expression group in line with a cutoff value of 25%. The somatic mutation data were calculated using the Maftools package and visualized using a waterfall diagram.

### 2.5 Gene enrichment analysis of CD44

The Correlation Analysis module of GEPIA was adopted for calculating correlation with target gene level using Pearson correlation method. The TOP 100 genes associated with CD44 in 33 types of cancer were obtained from GEPIA with the similar genes detection function, and Gene Ontology (GO) analysis was performed using these 100 genes. Corresponding genetic characteristics were constructed through CancerSEA (http://biocc.hrbmu.edu.cn/CancerSEA) database, besides, correlation analysis of target genes with 14 cancer functional states in different cancers was performed using gene set variation analysis (GSVA) algorithm. In addition, we obtained the HALLMARK gene set in Molecular Signatures Database (MSigDB) (http://software.broadinstitute.org/gsea/msigdb/index.jsp) database, and conducted gene set enrichment analysis (GSEA) of cancers through clusterProfiler package of R. The false discovery rate (FDR) and normalized enrichment score (NES) were determined in diverse biological processes of each cancer type.

### 2.6 Correlation between CD44 expression and tumor immunity

We adopted CIBERSORT method for calculating relative scores of 22 kinds of immune cells within 33 types of cancer, and the Spearman’s correlation of CD44 level with immune infiltration was further measured. Spearman’s correlation analysis was performed using TIMER2.0 database for analyzing the correlation between CD44 and immune checkpoints, chemokines, and receptor genes. In line with somatic data obtained based on TCGA database, we applied Maftools package in calculating TMB value and MSI score for each tumor. Then, the correlation of CD44 level with TMB or MSI was examined through Spearman’s correlation analysis.

### 2.7 Evaluation of the predictive role of CD44 in immunotherapy

TIDE (http://tide.dfci.harvard.edu/) has been developed as the computational framework for evaluating tumor immune escape potential according to gene levels in tumor samples. By comparing target genes with recognized immune response evaluation biomarkers [including TMB, MSI, CD274, CD8, interferon gamma (INFγ), and TIDE], the area under receiver operating characteristic (ROC) curve (AUC) was determined for assessing the ICB response prediction performance. Furthermore, we predicted the relation of gene level with clinical outcomes of ICB, and the changes in CD44 gene expression induced by ICB therapy were identified on the basis of immunotherapy cohorts of homologous *in vivo* mouse models and *in vitro* cell models obtained from Tumor Immune Syngeneic MOuse (TISMO) database (http://tismo.cistrome.org). Finally, the role of CD44 in clinical response of anti-PD-L1 immunotherapy was predicted using the GSE91061 dataset.

### 2.8 Single-cell profiling of CD44 expression in multiple cancers

10x Genomics data of three patients were read from single-cell datasets of six types of cancer using R Seurat package (4.2.3) to process data to construct Seurat objects, integrated with Harmony to eliminated batch effects. The low-quality cells were filtered according to the quality control criteria: cells with gene <200 & gene >5000, more than 15% derived from the mitochondrial genome UMI. The FindVariableFeatures function was used to select 2000 highly variable genes for PCA analysis and dimensionality reduction. The average CD44 expression of each cancer in each cell subpopulation was calculated and displayed by a heatmap.

### 2.9 Developmental trajectories of CD44 gene expression

Monocle2 was used to explore the developmental trajectories of CD44 gene expression during differentiation in T cells, B cells, and myeloid lineages. The UMI matrix was read from the seurat object, and then the monocle object was created using the newCellDataSe function with the parameter expressionFamily=negbinomial.size. Further, the DDRTree method of reduceDimension function was used to reduce dimension, and the cells sequentially ordered by orderCells function were visualized.

### 2.10 CellChat analysis of the communication profiles of CD44^+^ and CD44^-^ monocytes

Monocytes with CD44 gene expression > 0 was defined as CD44^+^ monocytes. Conversely, equal to 0 is defined as CD44^-^monocyte. The weights of interactions of CD44^+^ monocytes or CD44^-^ cells with other cells were calculated by CellChat (1.1.3) computeCommunProbe function. Further, the netAnalysis_signalingRole_scatter function was used to identify the signals that contribute most to the communication patterns and outgoing or incoming signals of certain cell populations. The function netVisual_bubble was used to identify key receptor-ligand pairs mediating intercellular communication.

### 2.11 Cell culture

In this work, we purchased human THP-1 monocytes and human HCT-116 CRC cell in Procell Life Science & Technology Co., Ltd. (China) Cells with STR identification and mycoplasma detection and cultivated them within RPMI-1640 medium that contained 10% fetal bovine serum (FBS) in an incubator with 5% CO2 under 37°C. 0.05 mM β-mercaptoethanol was further added into the THP-1 cell culture medium.

### 2.12 Macrophage polarization

THP-1 cells (1×105/well) were inoculated in the 24-well plate, followed by 24-h exposure to 100 ng/ml phorbol 12-myristate 13-acetate (PMA) (Abcam, ab120297) for differentiation induction to M0 macrophages. Then, lipopolysaccharide (100 ng/ml, LPS) (Sigma Aldrich, L2630) as well as interferon-gamma (20 ng/ml, IFN-γ) (Novoprotein, GMP-CI57) was added to induce differentiation into M1 macrophages. M0 macrophages were also exposed to interleukin (IL)-4 (20 ng/ml, Sigma Aldrich, SRP3093-20UG) together with IL-13 (20 ng/ml, Sigma Aldrich, SRP3274) for 24 h to induce differentiation into M2 macrophages.

### 2.13 Small interfering RNA transfection

siRNAs specifically targeting CD44 were prepared in Tsingke Biotechnology (China). Cells (3 × 10^5^/well) were seeded into 6-well plates till reaching reach 30–40% density, followed by transfection using TSnanofect V2 transfection reagent in line with specific protocols (Tsingke Biotechnology, China). **Supplementary Table S1** displays siRNA sequences. The transfection concentration of siRNA was 50 nM, that is, if the total volume of each well was 1 mL, the system consisted of 800 μl antibiotic-free cell culture medium + 2.5 μl siRNA diluted with 100 μl Opti-MEM +2 μl transfection reagent diluted with 100 μl Opti-MEM. After transfection, the cells were cultured in an incubator with 5% CO2 at 37°C for 24 hours, and then replaced with fresh medium. The transfection efficiency of FAM negative control was observed by fluorescence microscope. When the fluorescence positive cells exceeded 80%, qRT-PCR was used to detect the target gene expression.

### 2.14 Quantitative real-time PCR

TRIzol reagent (Gene, China) was used for extracting total RNA from cultured cells. The concentration and purity of RNA were evaluated using Nanodrop (Thermo,USA). The CDNA was synthesized using the Takara PCR Thermal cycler (Takara,Japanese), according to the instructions of HiScript III 1st Strand cDNA Synthesis Kit (Vazyme Biotech, China) with the reaction condition at 37°C for 15 min and 85°C for 5 sec. The total amount of RNA was 1μg.QRT-PCR were performed using Bio-rad T100 Real-Time PCR system (Bio-rad, USA) according to the manufacturer’s instructions of HiScript II Q RT SuperMix for qPCR (Vazyme Biotech, China). The reaction system was: 10 μ SYBR qPCR Master Mix, 0.5 μl forward Primer (10 µM) and 0.5 μl reverse Primer (10 µM) and 1μl CDNA template. The reaction conditions used was: stage 1 pre-denaturation, 95°C for 30s; stage 2 cycling reaction, performed with 40 cycles for 95° C 10 s,60° C,20s. The primer sequences were listed in **Supplementary Table S1**. The relative mRNA expression levels of target genes was calculated by 2^-ΔΔCt^ approach.

### 2.15 Cell counting kit-8 assay

HCT116 cells (5×10^3^/well) were inoculated into the 96-well plate. At 0, 24, 48, 72, and 96 h after treatment, CCK-8 reagent (10 μl, Dojindo, Japan) and RPMI 1640 medium (90 μl) were introduced into every well. After 1-h incubation under 37°C and 5% CO2, absorbance values were measured at 450 nm.

### 2.16 Colony formation assay

We inoculated control or transfected HCT-116 cells (800 cells/well) into a 6-well plate prior to another 14-day incubation within 2 mL medium under 37°C with 5% CO2. Thereafter, those colonies formed were immersed in 4% paraformaldehyde for a 20-min period and washed with PBS before 10-min staining using 0.1% crystal violet. Finally, colony counts of different groups were determined.

### 2.17 Wound-healing assay

Three parallel lines were pre-marked on the back of 6 well cell culture plates before cell inoculation. HCT 116 or cells transfected with siRNA were seeded in 6-well cell culture plates with 1×10^6^ cells/well till reaching 90% density. Cells were then scraped with the 200-μl pipette tip perpendicular to the marker line, and cellular fragments was washed with PBS. After that, cultures were changed to serum-free medium for 24 hours. At 0 and 24 h, the degree of scratch healing at the same scratch was observed under an inverted microscope. Image J was then adopted for measuring and analyzing wound width, and migration rate was calculated. Wound healing rate = (0 h-24 h wound width)/0 h wound width ×100%.

### 2.18 Transwell assay

We re-suspended HCT-116 cells (5×10^4^/well) into serum-free RPMI-1640 medium (200 μl) and seeded them into top Transwell chambers (8μm), which were coated with matrix gel (BD Biosciences, USA), and contained RPMI 1640 medium (600 μl) with 10% FBS in the bottom chambers. In addition, in the co-cultured transwell system, macrophages under different treatment conditions were incubated in the bottom chambers with 10% FBS RPMI 1640 medium. After 48h co-culture, we immersed cells on the membrane bottom in the Transwell chamber in 4% formaldehyde prior to staining using 0.1% crystal violet. While cotton balls were used to remove cells on top of the membrane. A microscope was utilized for image acquisition.

### 2.19 Western blot

Proteins from cells were extracted using RIPA lysis buffer (Solarbio,R0020). BCA Protein Assay Kit (Beyotime, P0012S) was used to quantify protein concentrations. The protein was separated by 10% or 15% SDS-PAGE at 80V voltage. After the markers was separated, the voltage was changed to 120V.Afterwards the protein on PAGE gel were electrotransferred to a PVDF membrane at 200 mA for 1 h. The PVDF membrane was blocked with 5% milk blocking solution for 2 hours. Then, PVDF membrane were incubated with the diluted primary antibodies overnight at 4 ° C. The antibodies used in this study were listed in **Supplementary Table S2**. After washing with PBST, the PVDF membrane was incubated with a 1:1000 diluted second antibody conjugated with HRP for 1 hour. Exposure was performed under the Tanon5200 automatic chemiluminescence imaging system. The density of bands were analyzed using image J,normalized with GAPDH levels.

### 2.20 Statistical analysis

R 4.2.3, SPSS v26, and GraphPad Prism 8.0 were employed for statistical analysis. Two group data in normal distribution and homogeneity of variance were compared by student’s t-test (two-tailed). Variables in non-normal distribution were analyzed by Wilcoxon rank sum test. Multiple groups were compared by Kruskal-Wallis test and one-way analysis of variance (ANOVA).

## 3 Results

### 3.1 CD44 levels within normal and cancer samples

**Figure 1:**
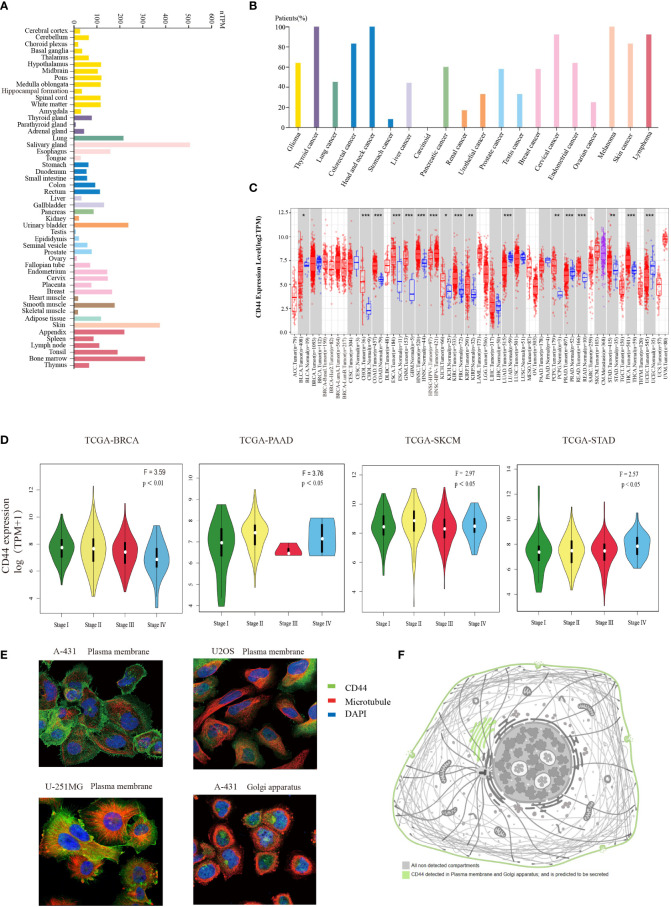
Expression profiles of CD44 in normal and tumor tissues. **(A)** Bar plot of CD44 gene expression profiles using TPM in a variety of normal tissues from the HPA database. **(B)** Percentage of patients with moderate to strong membranous immunoreactivity of CD44 in the total number of patients with a certain cancer,summary of pathological analysis based on HPA database. **(C)** Box plot of comparation of CD44 gene expression in different tumors and normal tissues using log_2_TPM from the TIMER2.0 database (* P < 0.05; ** P < 0.01; *** P < 0.001). **(D)** Violin plots showing the expression levels of CD44 in different pathological tumor tissues. **(E)** Immunofluorescence staining of subcellular localization of CD44 in A-431, U2OS, and U-251MG cells obtained from HPA database. **(F)** Pattern graph of the subcellular localization of CD44 obtained from the HPA database.

To determine CD44 levels within normal and cancer samples, we investigated CD44 gene expression pattern in pan-cancer based on publicly available gene expression data. Firstly, the CD44 mRNA expression within different non-carcinoma samples was analyzed through HPA database. As a result, CD44 showed wide expression within various tissues, and was highly expressed in salivary gland, skin, bone marrow, pancreas, and bladder tissues (**Figure 1A**). According to the immunohistochemical analysis of the expression level of CD44 in cancer from the HPA database, the study found that CD44 exhibited moderate to strong membranous immunoreactivity, often accompanied with weak cytoplasmic staining, in a few cases of most cancer types. Most melanoma and cervical cancer cases were strongly stained. (**Figure 1B**). Further, TIMER2.0 platform was adopted for analyzing differential CD44 expression within cancer and non-carcinoma samples from TCGA dataset. Compared with normal tissues, CD44 was markedly up-regulated within many cancer tissues, including cholangiocarcinoma (CHOL), colon adenocarcinoma (COAD), esophageal carcinoma (ESCA), glioblastoma multiforme (GBM), HNSC, kidney chromophobe (KICH), kidney renal clear cell carcinoma (KIRC), kidney renal papillary cell carcinoma (KIRP), pheochromocytoma and paraganglioma (PIPG), rectum adenocarcinoma (READ), stomach adenocarcinoma (STAD), and thyroid carcinoma (THCA) cohort tissues. However, CD44 expression apparently decreased within bladder urothelial carcinoma (BLCA), lung adenocarcinoma (LUAD), prostate adenocarcinoma (PRAD), and uterine corpus endometrial carcinoma (UCEC) tissues (**Figure 1C**). Additionally, the CD44 expression in diverse cancer pathological stages was analyzed based on GEPIA database, as a result, CD44 expression related to the clinical pathological stages of breast invasive carcinoma (BRCA), pancreatic adenocarcinoma (PAAD), skin cutaneous melanoma (SKCM), and STAD (**Figure 1D**). Based on HPA immunofluorescence staining of subcellular localization, CD44 protein was strongly stained on the plasma membrane of A-431, U2OS, and U-251MG cells (**Figure 1E**), and CD44 protein was also detected on the Golgi apparatus and extracellularly secreted (**Figure 1F**).

### 3.2 Prognostic prediction of CD44

**Figure 2:**
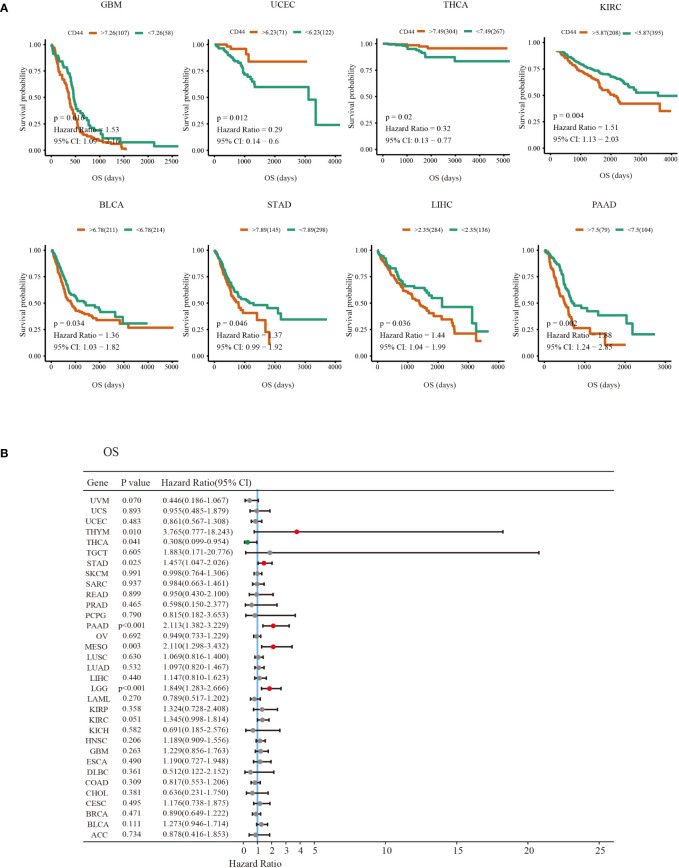
Prognosis value of CD44 in different types of cancer. **(A)** Kaplan-Meier survival curves of the OS outcomes of CD44 high expression (red) and low expression groups (green)in various cancers. **(B)** Forest plots of univariate Cox regression analyses of the prognostic role of CD44 in different types of cancer OS survival.Hazard Ratio (HR)>1 indicates that CD44 is a risk factor for prognosis (red dot). HR<1 indicates that CD44 has a protective effect on patient prognosis (green dot); A P value >0.05 indicates no statistical significance (gray dots).

Correlation between CD44 expression and OS as well as DSS was evaluated through univariable COX regression and Kaplan-Meier survival analyses. As suggested by the forest plot for univariable COX regression, high CD44 expression was related to dismal OS in thymic epithelial neoplasms(THYM), STAD, PAAD, and MESO brain lower grade glioma (LGG) [hazard ratio (HR)>1, P<0.05), whereas CD44 served as the good prognostic factor for OS in THCA (HR<1, P<0.05) (**Figure 2B**). Additionally, CD44 level was related to adverse DSS of BLCA, KIRC, HNSC, PAAD, STAD and LGG (HR>1, P<0.05), but CD44 was a good prognostic factor for DSS of BRCA cohort (HR<1, P<0.05) (**Supplementary Figure S2B**). As shown by the Kaplan-Meier survival analysis, CD44 up-regulation was indicative of shortened OS and DSS of GBM, BLCA, STAD, liver hepatocellular carcinoma (LIHC), PAAD, and KIRC, while it was associated with prolonged OS or DSS in UCEC, THCA and BRCA (**Figure 2A**; **Supplementary Figure S2A**).

### 3.3 Genome alterations and mutation profiles of CD44

**Figure 3:**
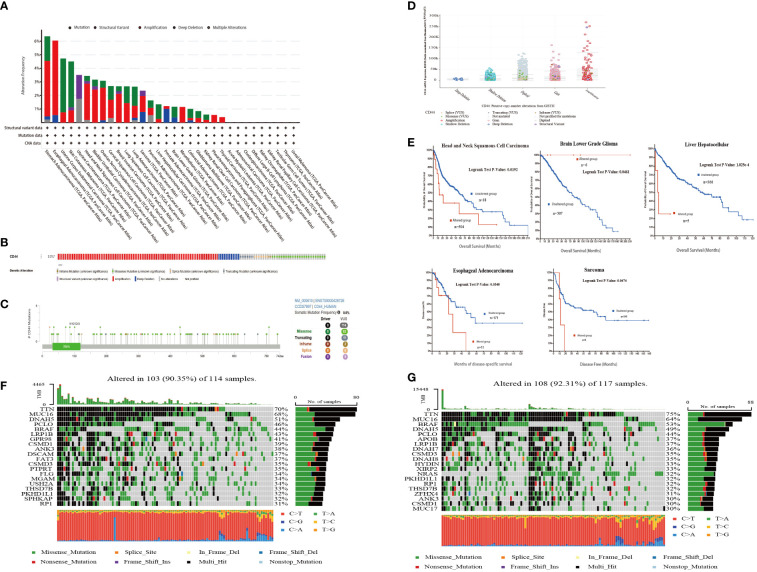
CD44 mutation landscape in pan-cancer of TCGA. **(A)** Bar plot of genetic alteration characteristics of CD44 in 32 different tumors from TCGA database;mutation(green),structuralvariant(purple),amplification(red),deep deletion(blue),multiple alterations(gray). **(B)** OncoPrint visual summary of types and overall proportion of genetic alterations of CD44 in pan-cancer from cBioPortal. **(C)** Protein domain diagram of CD44 mutation type, number and sites. **(D)** Box plot of CD44 expression levels of different types of gene mutations. **(E)** Kaplan-Meier curves of patients with different cancers from TCGA with CD44 altered group(red) and CD44 unaltered group(blue). **(F)** The genomic somatic mutation profiles of the CD44 low expression group in the TCGA-SKCM cohort. **(G)** The genomic somatic mutation profiles of the CD44 high expression group in the TCGA-SKCM cohort.

Next, the cBioPortal database was used to analyze the overall genome alternations of CD44 in 10,967 samples from 32 types of cancer in TCGA. The types of CD44 mutations mainly included amplification, mutation, and structural variation. The mutation rate of CD44 in STAD and ESCA was the highest (>6%), and the main mutation type was amplification. In addition, CD44 had the highest proportion of structural variations in UCEC (**Figure 3A**). In the mass, the main mutation type of CD44 in pan-cancer was amplification, and the overall genome alternation rate of CD44 in pan-cancer was 2.2% (**Figure 3B**). The mutation types, quantities, and sites of CD44 gene alternations were explored using the cBioPortal tool. CD44 presented 114 mutations with 0~742 amino acids, and the frequency of somatic mutation was 0.9%. Among them, missense mutation was predominant, with a total of 83 mutation sites and 9 fusion mutation sites (**Figure 3C**). By examining the effect of different types of gene copy number alterations (CNAs) on CD44 gene expression, amplification was the most common and was associated with an increase in CD44 expression (**Figure 3D**). To investigate the association between CD44 gene mutations and alternations and clinical outcomes in, we found that CD44 gene mutations were associated with shortened OS (log-rank P=0.0192),as well as shortened DFS (log-rank P=0.0340) in esophageal adenocarcinoma and shortened DSS (log-rank P=0.0474) in sarcoma (**Figure 3E**). Furthermore, in TCGA-SKCM cohort, diversity of mutation profile in the CD44 high expression group and the CD44 low expression group were observed.GPR98, DSCAM, FAT3, PTPRT, FLG, MGAM, USH2A, and SPHKAP had higher mutation rates in the CD44 ^high^group, while DNAH7, DNAH8, HYDIN, XIRP2, NRAS, ZFHX4, and MUC17 had higher mutation rates in the CD44 ^low^ group. Moreover, the CD44^high^ group had decreased BRAF somatic mutation frequency but increased CSMD1 and ANK3 somatic mutation frequencies compared to the CD44 ^low^ group (**Figures 3F, G**).

### 3.4 Functional enrichment analysis of CD44 in pan-cancer

**Figure 4:**
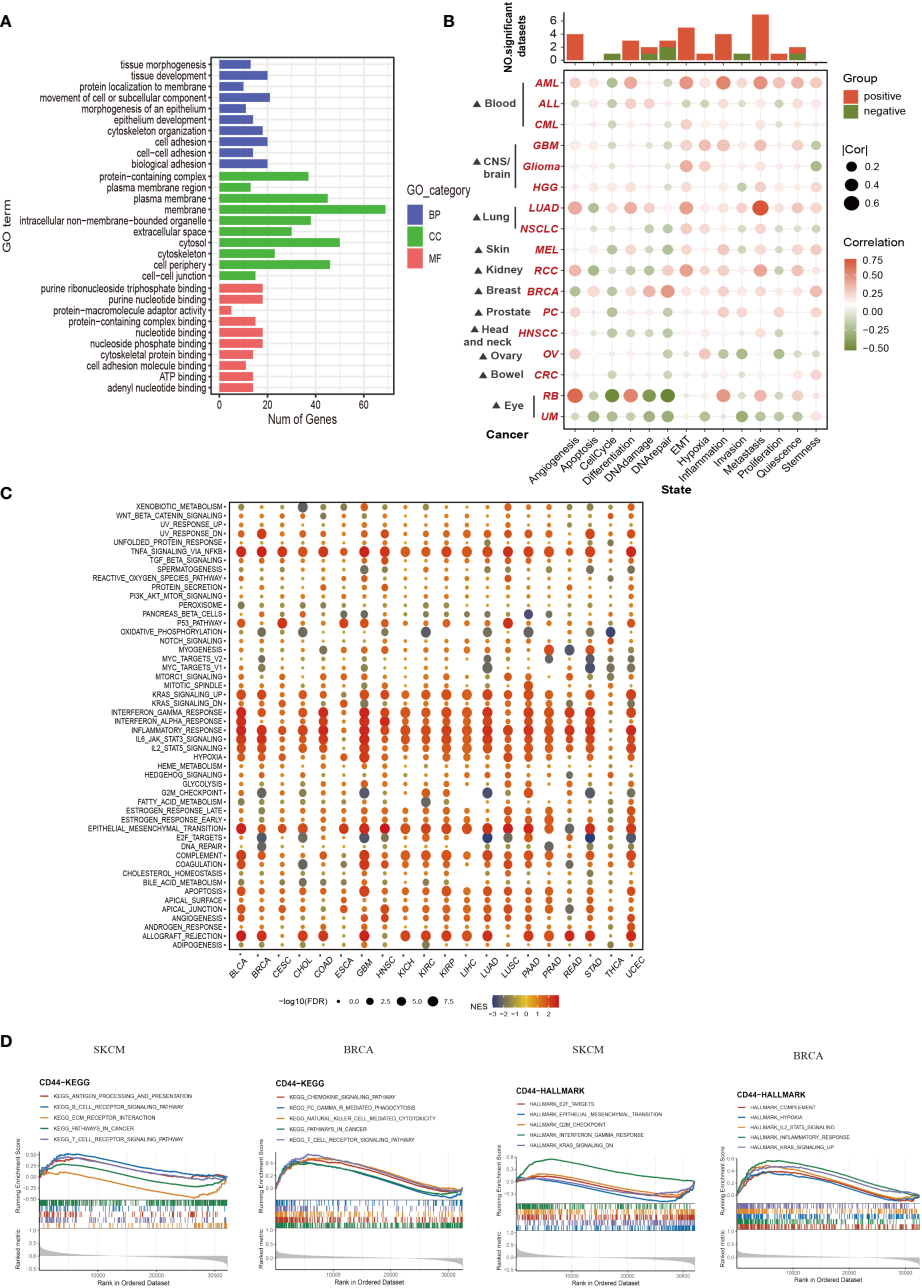
Functional enrichment analysis of CD44 in pan-cancer. **(A)** GO enrichment analysis of the TOP 100 genes associated with CD44 in 33 types of cancer obtained from GEPIA. Blue, red and green respectively represent biological process(BP),cellular component **(CC)** and molecular function(MF). **(B)** The association between CD44 and 14 kinds of cancer functional state from the CancerSEA database. **(C)** Bubble plot of the Hallmark GESA of CD44 in pan-cancer. **(D)** Enrichment analysis of CD44 with KEGG and Hallmark gene sets in TCGA breast cancer(left) and melanoma cohorts(right).

For exploring the CD44 molecular mechanisms affecting cancer occurrence biological processes, we performed pathway enrichment analysis on CD44 and its associated genes. GO enrichment analysis was conducted on 100 genes screened by GEPIA2 with the highest correlation with CD44 in pan-cancer. These genes were involved in the following biological processes: cell adhesion, nutrient binding, organelle formation, and tissue organ formation (**Figure 4A**). The relation of CD44 with 14 functional states within pan-cancer was analyzed based on CancerSEA database. As a result, CD44 was closely related to tumor-promoting biological processes, such as metastasis, angiogenesis and epithelial-mesenchymal transition (EMT) of AML, LUAD, and RCC (**Figure 4B**). To clarify the specific signaling pathways regulated by CD44, we performed GSEA and confirmed that CD44 mainly participated in inflammatory responses, interferon, interleukin, EMT, and KRAS signaling pathways in pan-cancer (**Figure 4C**). Further enrichment analysis of CD44 with Kyoto Encyclopedia of Genes and Genomes (KEEG) and Hallmark gene sets in breast cancer and melanoma cohorts from TCGA showed that CD44 was involved in the following immune-related pathways in melanoma: antigen processing and presentation; B cell receptor signaling pathway; T cell receptor signaling pathway; and IFNγ response. CD44 was involved in the chemokine signaling pathway, Fc receptor-mediated phagocytosis, natural killer (NK) cell-mediated cytotoxicity, and T cell receptor signaling pathway in breast cancer (**Figure 4D**). Therefore, these findings indicated that CD44 is crucial for tumor progression and immune regulation.

### 3.5 CD44 correlation with immune cell infiltration and key immune regulators

**Figure 5:**
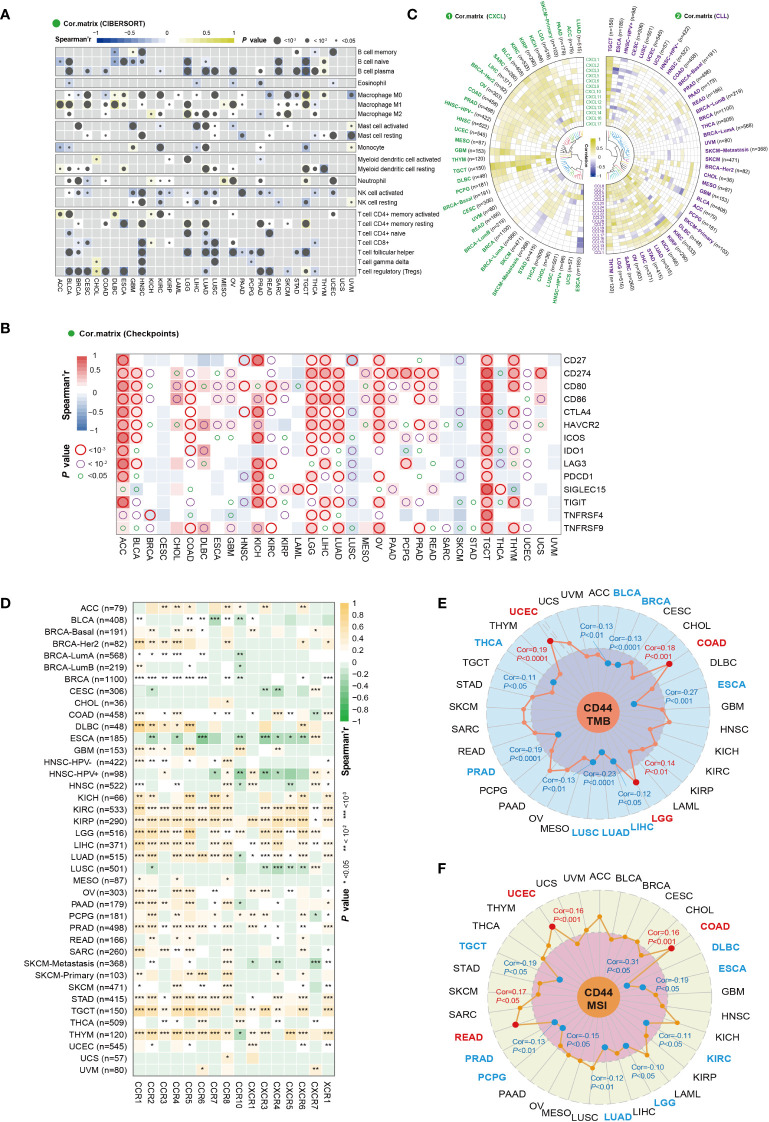
CD44 correlation with immune cell infiltration and key immune regulators. **(A)** Correlation analysis between CD44 and immune cell infiltration by CIBERSORT analysis in pan-cancer. Spearman’s correlation coefficient, blue indicates negative correlation, and yellow indicates positive correlation. **(B)** Correlation analysis of CD44 expression with immune checkpoints and costimulatory factors in pan-cancer. Spearman’s correlation coefficient, blue indicates negative correlation, and red indicates positive correlation. **(C)** Correlation analysis of CD44 and chemokines in pan-cancer. Pearson’s correlation coefficient, blue indicates negative correlation, and yellow indicates positive correlation. **(D)** Correlation analysis of CD44 and chemokine receptor in pan-cancer. Spearman’s correlation coefficient, green indicates negative correlation, and orange indicates positive correlation. **(E)** Correlation analysis between CD44 and TMB in pan-cancer. Pearson’s correlation coefficient, blue indicates negative correlation, and yellow indicates positive correlation. **(F)** Correlation analysis between CD44 and TMB in pan-cancer. Pearson’s correlation coefficient, blue indicates negative correlation, and yellow indicates positive correlation.

For exploring the effect of CD44 on TME, CIBERSORT deconvolution algorithm was adopted for calculating overall correlation landscape of CD44 with immune cell infiltration in 33 cancer types. The results indicated that CD44 exhibited significant heterogeneity in infiltrating degrees of immune cells within diverse cancer types. In general, however, CD44 was positively related to infiltration of macrophages, neutrophils, and CD4^+^ memory T cells, but negatively related to T follicular helper cells, B cells, NK cells, and regulatory T cells (Tregs) in most cancers. From the perspective of cancer types, CD44 was most significantly positively related to immune cell infiltration within testicular germ cell tumors (TGCTs) (**Figure 5A**). Spearman’s correlation analysis revealed that CD44 was correlated with 14 major immune checkpoints and co-stimulating factors within diverse cancer types. CD44 showed positive relation to the sub-immune checkpoint in most cancers, including TGCTs, adrenocortical carcinoma (ACC), KICH, LGG, LIHC, LUAD, and ovarian serous cystadenocarcinoma (OV) (**Figure 5B**).

Because chemokines have important effects on immune cell migration into cancers, we investigated the effect of CD44 on chemokines and chemokine receptors. The correlation of CD44 with the main chemokines of the CXC subfamily and CC subfamily was analyzed, and a heatmap was used to visualize chemokines with a Pearson correlation coefficient of P<0.05. In most cancers, CD44 was positively correlated with chemokines of the CXC subfamily, while CD44 was negatively correlated with most chemokines in ESCA. In addition, CD44 was negatively correlated with multiple chemokines of the CC subfamily in the HNSC-HPV^+^ and ESCA cohorts (**Figure 5C**). CD44 was positively correlated with most chemokine receptors in KICH, KIRC, KIRP, LGG, LIHC, LUAD, STAD, TGCT, and THYM. On the contrary, CD44 showed negative relation to most chemokine receptors within ESCA and LUSC (**Figure 5D**).

TMB and MSI represent two important biomarkers used to predict the ICIs therapeutic effect. CD44 expression showed positive relation to the TMB values within COAD, LGG, and UCEC, but negative relation to the TMB levels within BLCA, BRCA, ESCA, LIHC, LUAD, LUSC, PRAD, and THCA (**Figure 5E**). Additionally, CD44 showed positive relation to the MSI values in COAD, READ, and UCEC, but negative relation to the MSI values of DLBC, ESCA, KIRC, LGG, LUAD, PCPG, PRAD, and TGCT (**Figure 5F**). On the whole, CD44 exerts an essential impact on TME, including immune checkpoints, immune cell infiltration, chemokines, MSI and TMB, suggesting that CD44 may be novel key target for immunotherapy.

### 3.6 Single-cell analysis of CD44 expression and differentiation trajectory

**Figure 6:**
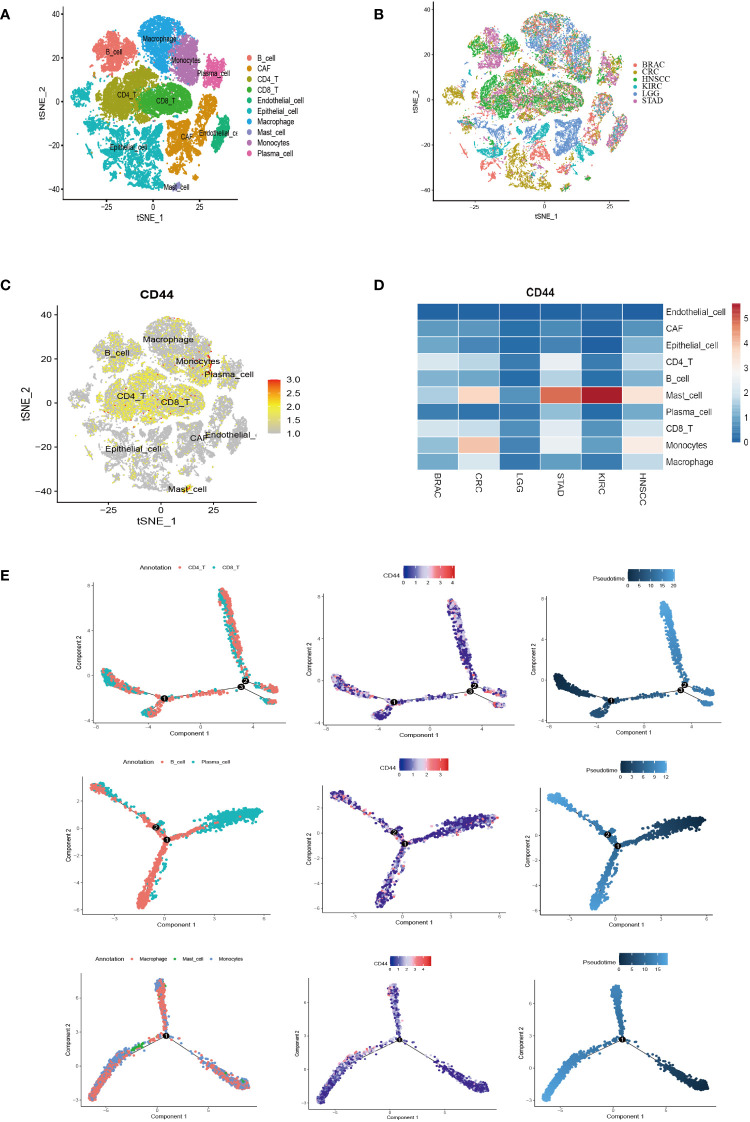
Single-cell analysis of CD44 expression distribution and differentiation trajectory in cell clusters of different types of cancer. **(A)** t-SNE plot of the major cell type clusters of six types of cancer BRAC, CRC, HNSCC, KIRC, LGG, and STAD. Each cell type is color-coded. **(B)** t-SNE plot showing the major cell clusters of BRAC, CRC, HNSCC, KIRC, LGG, and STAD. Cancer types are color-coded. **(C)** t-SNE plot of the expression and distribution of CD44 in the cell clusters of BRAC, CRC, HNSCC, KIRC, LGG, and STAD. Color scale represents gene expression level. **(D)** Heatmap of the expression of CD44 in different cell clusters of BRAC, CRC, HNSCC, KIRC, LGG, and STAD at a single-cell level. **(E)** Pseudotime analysis of the differentiation trajectory of CD44 in T lineage (top), B lineage (middle), and myeloid lineage (bottom).

Six were downloaded from GEO database containing 18 patients of six types of cancer. A total of 47,023 cells were obtained after performing the quality control process (**Supplementary Figure S4**). After reclustering, a total of ten major cell clusters were obtained as follows: three stromal cell types [endothelial cells (PECAM1), epithelial cells (EPCAM and KRT18), and cancer-associated fibroblasts (CAFs; COL1A1)]; seven immune cell types [CD4+ T cells (CD4), CD8+ T cells (CD8A), B cells (CD79A and MS4A1), plasma cells (JCHAIN), macrophages (CD68), monocytes (CD14 and FCGR3A), and mast cells (TPSB2)] (**Figure 6A**; **Supplementary Figure S4**). The cells derived from different tumor tissues and different datasets were evenly distributed and did not show obvious disease specificity (**Figure 6B**). This work examined CD44 expression and distribution in stromal cells and immune cells at the single-cell level. CD44 had a higher expression in immune cells compared to stromal cells (**Figure 6C**). Although CD44 was widely distributed in B cells and T cells, it had higher expression within monocytes and mast cells. Comparison of the expression differences of CD44 among different cell clusters in the six cancer types demonstrated that CD44 had the highest expression in mast cells of KIRC, STAD, HNSC and CRC. In addition, monocytes also had high expression of CD44 in HNSC and CRC. Intertumoral heterogeneity analysis indicated that CD44 had the broadest distribution of cell subsets in STAD (**Figure 6D**). According to Monocle2 analysis, CD44 gene expression altered depending on cell trajectory with the differentiation of various immune cells. In the T lineage development trajectory, CD44^high^ T cells were located at the end of each branch point of the development tree, while CD44^low^ T cells were located in the early CD4^+^ T cell cluster development and late CD8^+^T cell differentiation. However, expression of CD44 in the B lineage was evenly distributed throughout the cell development trajectory. With the differentiation trajectory of myeloid lineage cells, the expression of CD44 showed an increasing trend, especially in monocyte subsets (**Figure 6E**).

### 3.7 CD44 as an important regulatory factors in cell-cell communication

**Figure 7:**
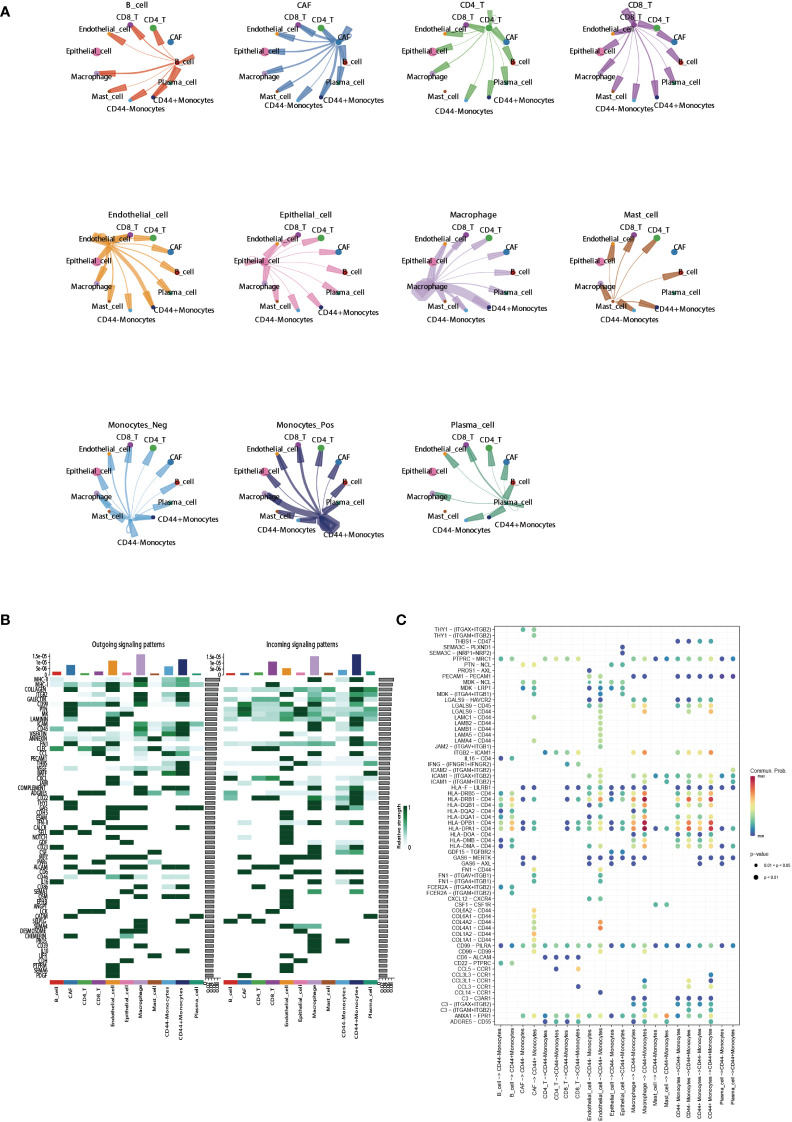
Regulatory role of CD44 in cell-cell communication. **(A)** Circle plots showing cell-cell communications of main cell clusters. Each cell cluster acts as a signaling sender or signaling receiver conducting intercellular crosstalk with CD44^+^monocytes and CD44^-^ monocytes, respectively. **(B)** Profile of incoming and outgoing information flows mediated by different signaling pathways in the main cell clusters. **(C)** Communication probabilities of important ligand-receptor pairs mediated the cell-cell communication from main cell clusters to CD44^+^monocytes or CD44^-^ monocytes. The color of the dot represents the probability of communication, and the size of the dot corresponds to p-value. The ligand receptor corresponding to empty meaning does not mediate communication in this cell.

To clarify the regulatory role of CD44 in cell-cell communication, we selected monocytes which have a high CD44 expression as the research object. CellChat was used to analyze the differences in signaling interaction patterns between CD44^+^ monocytes and CD44^-^ monocytes. When CD44^+^ monocytes acted as the receiver of *incoming signals*, the strength of communication increased when interacting with CAFs, epithelial cells, and macrophages (as signaling senders) compared to CD44^-^ monocytes (**Figure 7A**). When monocytes acted as the sender of outgoing signals, the interaction of CD44^+^ monocytes with CAFs or endothelial cells was more intense than that of CD44^-^ monocytes. Similarly, in the incoming and outgoing information flows mediated by different signaling pathways, CD44^+^ monocytes had a higher overall information flow intensity than CD44^-^ monocytes, including incoming signaling patterns mediated by MHC-II, CD22, CD23, CD45, and ICAM, as well as outgoing signaling patterns mediated by ICAM, ITGB2, CD45, CD86, and ADGRE5 (**Figure 7B**). In addition, ligand-receptor (L-R) pairs mediated by CD44 exhibited high activity in the interaction between endothelial cells and CAFs with CD44^+^ monocytes. In particular, L-R pairs formed by the combination of CD44 and the collagen family genes (COL1A1, COL1A2, COL4A1, COL4A2, COL6A1, and COL6A2), which were highly expressed in CAFs, were observed in the interaction between CAFs and CD44^+^ monocytes. In the signaling communication between endothelial cells and monocytes, the L-R pairs formed by CD44 and the adhesion protein gene family (LAMA4, LAMA5, LAMB1, LAMB2, and LAMC1) were also highly active in the CD44^+^ monocyte subgroup (**Figure 7C**).

### 3.8 Predictive ability of immunotherapy response based on CD44 expression

**Figure 8:**
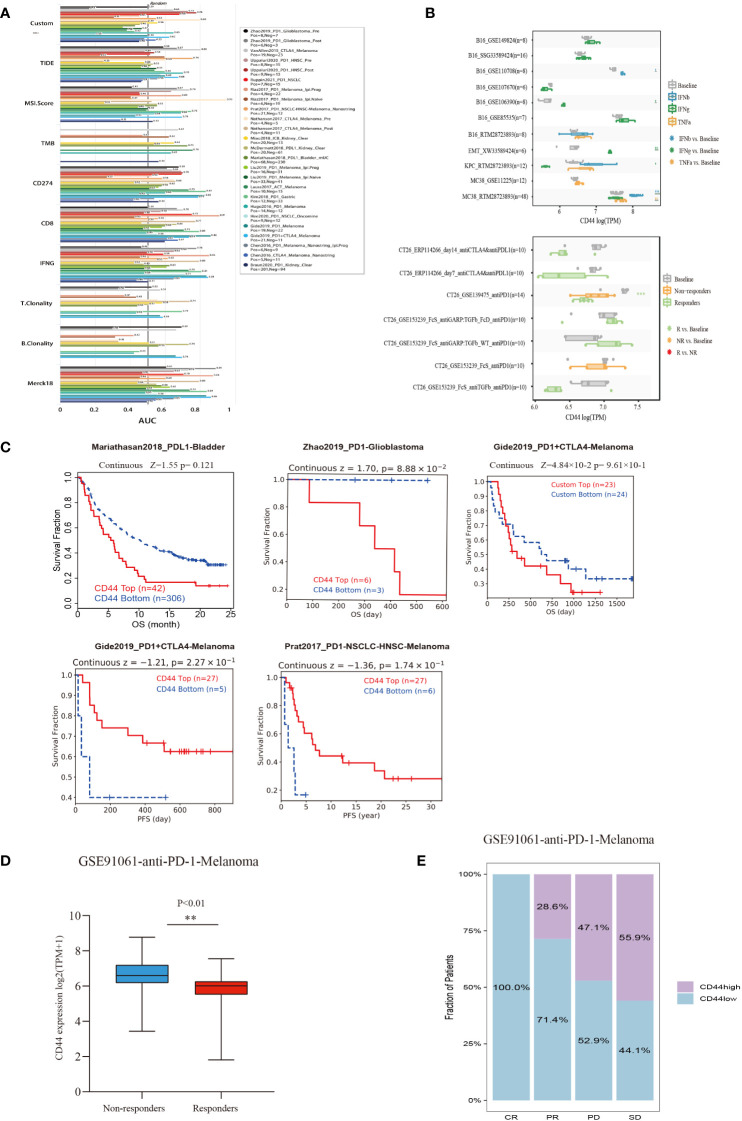
Evaluation of the predictive value of CD44 in cancers immunotherapy response using the ICB treatment clinical cohort from TIDE. **(A)** Bar plot of AUC from the TIDE model including 25 public cancer immunotherapy cohorts to predict ICB response prediction value of CD44. **(B)** Box plots of the gene expression of CD44 before and after cytokine therapy(IFNβ, IFNγ, and TNF-α)in cancer cell lines (above)and in syngeneic mouse CT26 tumor models before and after ICB therapy (anti-PD1 or anti-PD-L1) (blow)from TIMSO database. **(C)** Kaplan–Meier plots of patients with top half and bottom half CD44 expression levels, using the data of ICB therapy cohort of from the TIDE database. **(D)** Correlation between CD44 expression and anti-PD-1 immunotherapy response in the GSE91061 melanoma cohort. **(E)** Effect of CD44 on anti-PD-L1 immunotherapy on the clinical response rate in the GSE91061 melanoma cohort. ** p<0.01.

The TIDE database was used to evaluate the possibility of CD44 as a new predictive marker for ICB. Among the 25 immunotherapy cohorts, CD44 had an AUC of >0.5 in 10 cohorts and >0.7 in 3 cohorts (Uppaluri2020_PD1_HNSC_Pre, Uppaluri2020_PD1_HNSC_Pos, and Nathanson 2017_CTLA4_Melanoma_Pre). In the melanoma anti-CTLA4 cohort, the AUC of CD44 was 0.8, indicating a higher predictive ability than any other marker, except MSI (AUC = 0.9) (**Figure 8A**).

For the *in vitro* models, we analyzed the B16 (mouse melanoma), EMT6 (mouse breast cancer), KPC (mouse pancreatic cancer), and MC38 (mouse colon cancer cells) models treated with IFNβ, IFNγ, and TNF-α. Compared to baseline, there was in increase in CD44 expression with the following treatments in the specific models: IFNβ in the B16 and MC38 models; IFNγ in B16 and EMT6 models; and TNF-α in MC38 model. The *in vivo* mouse model allowed analysis of ICB treatment, in which the responders of the CT26 anti-PD1 (GSE139475) cohort had significantly decreased CD44 expression compared to baseline but had no significant difference in CD44 expression compared to non-responders (**Figure 8B**). Moreover, we investigated the impact of CD44 on immunotherapy effect on immunotherapy cohorts in TIDE database. In Zhao2019_PD1-Glioblastoma and Gide2019_PD1+CTLA4-Melanoma cohorts, high-CD44-expression patients exhibited the unfavorable OS relative to low-CD44-expression counterparts, representing a worse immunotherapy outcome. However, high-CD44-expression patients exhibited an extended PFS compared with low-CD44-expression patients in Gide2019_PD1+CTLA4-Melanoma and Prat2017_PD1-NSCLC-HNSC-Melanoma cohorts (**Figure 8C**). Further, we verified the value of CD44 as a predictor for ICB efficacy, as a result, CD44 down-regulation was related to better immunotherapy response in a melanoma cohort undergoing anti-PD-1 therapy (GSE91061), and CD44 expression of non-response group markedly elevated (**Figure 8D**). As for CR (complete response) group, the proportion of low-CD44-expression patients was 100%, while those in the PR (partial Response), PD (progressive disease), and SD (stable disease) groups increased to 28.6%, 44.1%, and 47.1%, respectively (**Figure 8E**).

### 3.9 CD44 promotes CRC cells proliferation, migration and invasion

**Figure 9:**
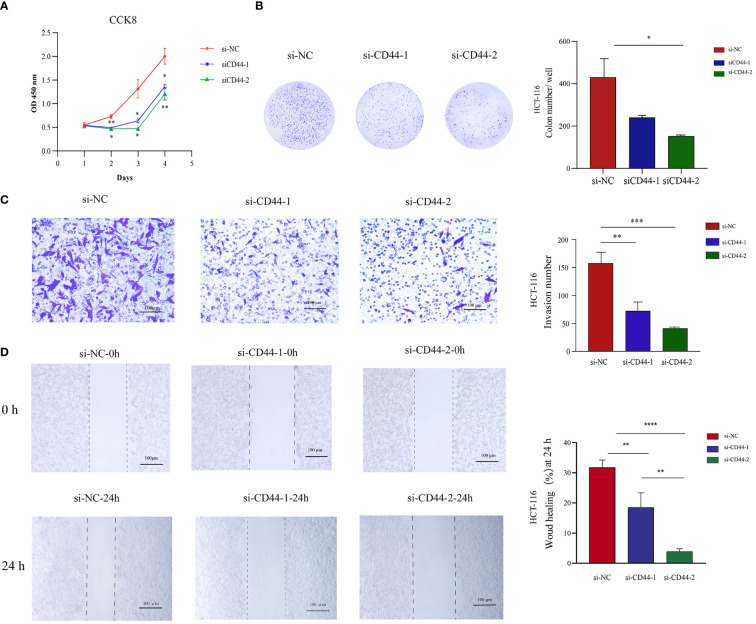
Effect of CD44 on the proliferation, invasion, and migration of CRC HCT-116 cells *in vitro.* **(A)** The effects of CD44 knowdown on proliferation of HCT-116 cells from 0 to 96 hour measured by CCK8 assays. **(B)** The effects of CD44 knowdown on colony formation of HCT116 cells. **(C)** The effects of CD44 knowdown on invasion capacities of HCT-116 cells evaluated by a transwell assay at 48 hours. **(D)** The effects of CD44 knowdown on migration capacities of HCT-116 cells an 0 and 24 hours determined by wound-healing assay. All experiments were performed in triplicate, and the representative data were presented as the mean ± SD. (*P < 0.05; **P < 0.01; ***P < 0.001, **** P < 0.0001).

Two independent small interfering RNAs (siRNAs) were prepared for silencing CD44 expression. CD44 siRNA was transfected into HCT-116 cells for a 24-h period. As demonstrated by qRT-PCR analysis, CD44 expression of CD44 siRNA2 group markedly decreased in comparison with negative control (NC) group (**Supplementary Figure S3A**). According to CCK-8 results, CD44 silencing markedly suppressed the proliferation of HCT-116 cells (**Figure 9A**). Similarly, colony formation assay revealed that knockdown of CD44 decreased colony formation of HCT-116 cells (**Figure 9B**). For exploring how CD44 affected HCT-116 cell migration and invasion, we conducted Transwell and scratch assays. Compared with NC group, CD44 knockdown inhibited HCT-116 cell migration and invasion (**Figures 9C, D**). Based on these results, CD44 down-regulation markedly inhibited HCT-116 cell growth, migration and invasion.

### 3.10 CD44 is crucial for maintaining M2 macrophage polarization and promoting CRC cell migration

**Figure 10:**
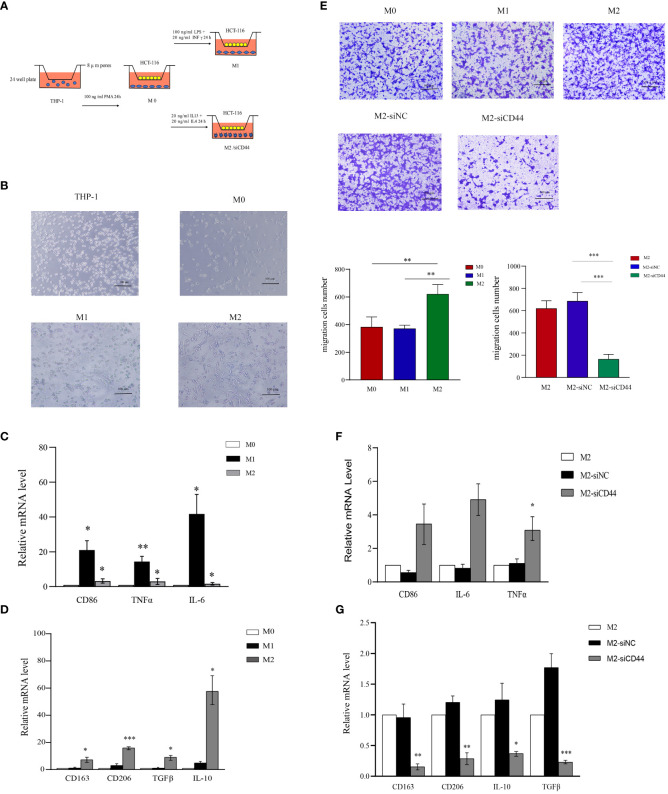
Knockdown of CD44 reducing M2 macrophage polarization and inhibiting the induction of CRC cell migration. **(A)** Experimental schematic diagram of THP-1 differentiate into different phenotypes of macrophage and co-cultured with HCT-116 in transwell to induce migration. **(B)** Morphological characteristics of THP-1 polarization into M0,M1 and M2 macrophages. **(C)** The relative mRNA expression of M1 markers (CD86, TNFα and IL-6) in M0, M1 and M2 macrophages determined by qPCR. **(D)** The relative mRNA expression of M2 markers (CD163, CD206, TGFβ and IL-10) in M0, M1 and M2 macrophages determined by qPCR. **(E)** The ability of M0,M1 and M2 macrophages to induce HCT-116 migration and the effect of CD44 knockdown on M2 macrophages on the inducing migration ability on HCT-116 cells measured by the transwell assay. **(F)** The effect of CD44 knockdown on the relative mRNA expression of marker genes of M1 macrophages in M2-like macrophages (CD86, TNFα and IL-6) determined by qPCR. **(G)** The effect of CD44 knockdown on the relative mRNA expression of marker genes of M2 macrophages in M2-like macrophages (CD163, CD206, TGF-β and IL-10) determined by qPCR. All experiments were performed in triplicate, and the representative data are presented as the mean ± SD, *P < 0.05, **P < 0.01, ***P < 0.001.

An *in vitro* model of macrophage polarization was constructed by first inducing THP-1 monocytes to differentiate into M0 macrophages using PMA and then incubating them with IFN-γ and LPS to differentiate them into classical M1-like macrophages or with IL-4 and IL-13 for differentiating in M2-like macrophages (**Figure 10A**). We conducted qRT-PCR for detecting macrophage markers levels in different phenotypes [M1 markers: CD86, tumor necrosis factor alpha (TNFα), and IL-6; M2 markers: CD163, CD206, transforming growth factor beta (TGFβ), and IL-10], confirming that macrophages with different phenotypes were successfully induced (**Figures 10B–D**). CD44 levels within diverse phenotype macrophages were analyzed, and CD44 expression significantly increased in M2 macrophages (**Supplementary Figure S3B**). For confirming the key effect of CD44 on maintaining M2 phenotype of macrophages, CD44 in M2-like macrophages was silenced (**Supplementary Figures S3C**, **S5A**). CD44 silencing significantly decreased the levels of CD163, CD206, TGFβ, and IL-10 in M2-like macrophages (**Figure 10G**). Knockdown of CD44 increased the expression of TNF-α in M2-like macrophages, however, the change of CD86 and IL-6 was not statistically significant (**Figure 10F**). Western blot assay comes to the similar conclusion (**Supplementary Figure S5B**).These results indicated that CD44 is essential for maintaining M2-like macrophage polarization. In the macrophage and CRC cell co-culture system, M2 macrophages significantly enhanced the migration of CRC cells compared to M0 and M1 macrophages, but this migration was significantly inhibited after knockdown of CD44.

## 4 Discussion

This work systematically detected CD44 expression and mutation profile in pan-cancer, as a result, CD44 expression significantly increased within various cancers and associated with poor prognoses, indicating that CD44 has extensive carcinogenic roles in cancer. Consistent with the present findings, CD44 expression is associated with poor prognostic outcome of glioma (38, 39), BLCA (40), STAD (41, 42), HNSCC (43), KIRC (44), LIHC (45), PAAD (46),MESO (47),and THYM (48).

Currently, most studies focus on CD44 as a surface marker of CSCs to promote tumor development and induce chemotherapy resistance through self-renewal and EMT pathways (49, 50). However, the immunoregulatory role of CD44 in the tumor microenvironment remains unclear.

Immune cell infiltration, soluble mediators and cellular receptors in TME play key roles in influencing tumorigenesis, regulating cancer progression, and regulating the immune response of cancer patients. Ma et al. confirmed that tumor-associated macrophages (TAMs) infiltration was related to the CD44 level within renal cell carcinoma cells (25). Gomez et al. proposed that CD44 regulated the effect of macrophages/monocytes in regulating head and neck CSCs. Additionally, they also demonstrated that recruiting monocytes increases the invasiveness of tumor cells via monocyte-activated CD44–VCAM-1 binding (51). Importantly, Ekaterina et al. effectively knocked out CD44 within myeloid cells, endothelial cells and astrocytes in mice and confirmed the role of CD44 expression in myeloid cells in promoting glioma invasion (52). In addition, Witschen et al. found that CD44 deficiency in breast cancer cells delays tumorigenesis and local progression *in vivo*, accompanied by the reduction of invasive CD206^+^ macrophages (53). Similarly, our study demonstrated that CD44 showed positive relation to macrophage infiltration but negative relation to B cell, natural killer (NK) cell, and T cell follicular helper infiltration. However, Treg infiltration was negatively correlated with CD44 in most cancers. In our previous single-cell analysis of the TME in CRC, we found that in the CD44-enriched region of the TME of the colon, there was an increase in crosstalk between SPP1^+^TAM and Foxp3+regulatory T cells (Tregs), which may increase the immunosuppressive microenvironment of CRC (54). Therefore, we speculated that CD44 promotes the tumor immunosuppressive microenvironment by suppressing cytotoxic immune cells and promoting TAM infiltration.

For the first time, we described the distribution characteristics of CD44 in the differentiation process of immune cells by using the pseudotime analysis.Interestingly, the expression of CD44 increased in the myeloid lineage cells differentiation trajectory, which means that CD44 may have a potential regulatory role in the differentiation process of myeloid cells in tumors. Furthermore,the cellchat analysis confirmed that CD44^+^ monocytes had a higher overall information flow intensity than CD44^-^ monocytes.Therefore, we speculate that CD44 may play a pivotal role in regulation of myeloid lineage cells, especially monocytes or macrophages.

Further, our study confirmed that CD44 expression was significantly up-regulated in M2 macrophages by using models that induced THP-1 to differentiate M1 or M2 macrophages *in vitro*. Knockdown of CD44 expression in M2-like macrophages was accompanied by downregulation of M2 macrophage markers including CD163, CD206, TGF-β, and IL-10 expression. At the same time, the M1 macrophage marker TNF-α was up-regulated, however, the change of M1 type characteristic marker CD86 was not statistically significant, and similar results were observed for IL-6.In addition, we demonstrated through Transwell assay that compared with M0 and M1 macrophages, M2 macrophages can induce HCT-116 cell migration, and this effect was inhibited when CD44 was down regulated. Therefore, we hypothesized that CD44 was an essential gene in maintaining the polarization phenotype of M2 macrophages. Due to some limitations in the model, we could not confirm that knocking down CD44 induced M2 macrophages to be reprogrammed into M1-like macrophages.

In summary, compared with previous studies that considered CD44 as a marker of CSCs, our study speculated that CD44 may promote the tumor immunosuppressive microenvironment by affecting myeloid cells in the TME, such as the differentiation of myeloid cells, the interaction between monocytes and other cells, the maintenance of M2 polarization which promote the progression of cancer.

The low response rate of patients to immune checkpoint blockade (ICB) therapy has become the main reason to limit its clinical use. Hence, screening biomarkers that can predict immune checkpoint inhibitors (ICIs) response in patients represents an urgent clinical issue to be addressed (55, 56). At present, the predictive value of PD-L1, MSI, TMB, and deficient mismatch repair (dMMR), and neoantigen as biomarkers for predicting ICB response has been widely accepted (57, 58). According to our results, CD44 showed positive relation to following factors: main immune checkpoints of testicular germ cell tumor (TGCT), ACC, KICH, LGG, LIHC, LUAD, and OV; TMB values of COAD, LGG, and UCEC; and MSI values of COAD, READ, and UCEC. Based on these positive correlations, CD44 expression may have a certain impact on treatment effect of ICB. Furthermore, we used TIDE database for evaluating CD44 prediction performance as an ICB response marker in 25 clinical immunotherapy cohorts. In part of the HNSC- PD1 and melanomaCTLA4 cohorts, CD44 as a marker of ICB response showed good predictive ability AUC > 0.7. In the anti-PD-1-melanoma cohort (GSE91061), CD44 up-regulation was associated with low response to PD-1 treatment. The conflicting conclusion on whether CD44 can serve as a prognostic indicator for immunotherapy in different immunotherapy datasets may be due to the heterogeneity of tumors. Because the prediction results in the present study were based on the publicly available clinical data on immunotherapy, CD44 as a marker of ICB response needs to be supported by larger sample size clinical trials.

## 5 Conclusion

The present study comprehensively explored the significance of CD44 in predicting prognosis of cancers as well as its effect on the immune microenvironment. Additionally, CD44 expression profile within the immune microenvironment at a single-cell level, pseudotime trajectory of the CD44 gene and its role in cell communication, function of CD44 in tumor cell growth and migration, and the effect on macrophage polarization were analyzed. In summary, anti-tumor therapy targeting CD44 holds great promise in improving and extending the survival of cancer patients.

## Data availability statement

The original contributions presented in the study are included in the article/**Supplementary Material**. Further inquiries can be directed to the corresponding author.

## Ethics statement

Ethical approval was not required for the studies on humans in accordance with the local legislation and institutional requirements because only commercially available established cell lines were used.

## Author contributions

QZ: Methodology, Writing – original draft, Writing – review & editing. XW: Formal analysis, Investigation, Software, Writing – review & editing. YL: Writing – review & editing. HX: Data curation, Methodology, Validation, Writing – review & editing. CY: Conceptualization, Funding acquisition, Investigation, Visualization, Writing – review & editing.

## References

[1] Tran KB Lang JJ Compton K Xu R Acheson AR Henrikson HJ. The global burden of cancer attributable to risk factors, 2010-19: a systematic analysis for the Global Burden of Disease Study 2019. Lancet. (2022) 400(10352):563–91. doi: 10.1016/S0140-6736(22)01438-6 PMC9395583 35988567

[2] Sunshine J. PD-1/PD-L1 inhibitors. Curr Opin Pharmacol. (2015) 23:32–8. doi: 10.1016/j.coph.2015.05.011 PMC4516625 26047524

[3] Wang D Wu X Sun YJ. Therapeutic targets and biomarkers of tumor immunotherapy: response versus non-response. Signal Transduct Target Ther. (2022) 7(1):331. doi: 10.1038/s41392-022-01136-2 36123348 PMC9485144

[4] Zhang Y Zhang ZJC. The history and advances in cancer immunotherapy: understanding the characteristics of tumor-infiltrating immune cells and their therapeutic implications. Cell Mol Immunol. (2020) 17(8):807–21. doi: 10.1038/s41423-020-0488-6 PMC7395159 32612154

[5] Bagchi S Yuan R. Immune checkpoint inhibitors for the treatment of cancer: clinical impact and mechanisms of response and resistance. Annu Rev Pathol Mech Dis. (2021) 16:223–49. doi: 10.1146/annurev-pathol-042020-042741 33197221

[6] Palmeri M Mehnert J Silk A Jabbour S Ganesan S Popli P. Real-world application of tumor mutational burden-high (TMB-high) and microsatellite instability (MSI) confirms their utility as immunotherapy biomarkers. Esmo Open. (2022) 7(1):100336. doi: 10.1016/j.esmoop.2021.100336 34953399 PMC8717431

[7] McGrail D Pilié P Rashid N Voorwerk L Slagter M Kok M. High tumor mutation burden fails to predict immune checkpoint blockade response across all cancer types. Ann Oncol. (2021) 32(5):661–72. doi: 10.1016/j.annonc.2021.02.006 PMC8053682 33736924

[8] Jardim D Goodman A de Melo Gagliato D Kurzrock R. The challenges of tumor mutational burden as an immunotherapy biomarker. Cancer Cell. (2021) 39(2):154–73. doi: 10.1016/j.ccell.2020.10.001 PMC7878292 33125859

[9] Xu Y Su G Ma D Xiao Y Shao Z Jiang Y. Technological advances in cancer immunity: from immunogenomics to single-cell analysis and artificial intelligence. Signal Transduct Target Ther. (2021) 6(1):312. doi: 10.1038/s41392-021-00729-7 34417437 PMC8377461

[10] Naor D Nedvetzki S Golan I Melnik L Faitelson Y. CD44 in cancer. Crit Rev Clin Lab Sci. (2002) 39(6):527–79. doi: 10.1080/10408360290795574 12484499

[11] Marhaba R Zöller M. CD44 in cancer progression: adhesion, migration and growth regulation. J Mol Histol. (2004) 35(3):211–31. doi: 10.1023/B:HIJO.0000032354.94213.69 15339042

[12] Chen C Zhao S Karnad A Freeman J. The biology and role of CD44 in cancer progression: therapeutic implications. J Hematol Oncol. (2018) 11(1):64. doi: 10.1186/s13045-018-0605-5 29747682 PMC5946470

[13] Guo Q Yang C Gao F. The state of CD44 activation in cancer progression and therapeutic targeting. FEBS J. (2022) 289(24):7970–86. doi: 10.1111/febs.16179 34478583

[14] Prochazka L Tesarik R Turanek J. Regulation of alternative splicing of CD44 in cancer. Cell Signal. (2014) 26(10):2234–9. doi: 10.1016/j.cellsig.2014.07.011 25025570

[15] Yan Y Zuo X Wei D. Concise review: emerging role of CD44 in cancer stem cells: A promising biomarker and therapeutic target. Stem Cells Transl Med. (2015) 4(9):1033–43. doi: 10.5966/sctm.2015-0048 PMC4542874 26136504

[16] Gomari M Farsimadan M Rostami N Mahmoudi Z Fadaie M Farhani I. CD44 polymorphisms and its variants, as an inconsistent marker in cancer investigations. Mutat Res Rev Mutat Res. (2021) 787:108374. doi: 10.1016/j.mrrev.2021.108374 34083044

[17] Williams K Motiani K Giridhar P Kasper S. CD44 integrates signaling in normal stem cell, cancer stem cell and (pre)metastatic niches. Exp Biol Med. (2013) 238(3):324–38. doi: 10.1177/1535370213480714 PMC11037417 23598979

[18] Dzobo K Sinkala M. Cancer stem cell marker CD44 plays multiple key roles in human cancers: immune suppression/evasion, drug resistance, epithelial-mesenchymal transition, and metastasis. Omics-a J Integr Biol. (2021) 25(5):313–32. doi: 10.1089/omi.2021.0025 33961518

[19] Hassn Mesrati M Syafruddin S Mohtar M Syahir AJB. CD44: A multifunctional mediator of cancer progression. Biomolecules. (2021) 11(12):1850–82. doi: 10.3390/biom11121850 PMC8699317 34944493

[20] Mattheolabakis G Milane L Singh A Amiji M. Hyaluronic acid targeting of CD44 for cancer therapy: from receptor biology to nanomedicine. J Drug Target. (2015) 23:605–18. doi: 10.3109/1061186X.2015.1052072 26453158

[21] Michalczyk M Humeniuk E Adamczuk G Korga-Plewko A. Hyaluronic acid as a modern approach in anticancer therapy-review. Int J Mol Sci. (2022) 24(1):103–32. doi: 10.3390/ijms24010103 PMC9820514 36613567

[22] Takeda M Ogino S Umemoto R Sakakura M Kajiwara M Sugahara KN. Ligand-induced structural changes of the CD44 hyaluronan-binding domain revealed by NMR. J Biol Chem. (2006) 281:40089–95. doi: 10.1074/jbc.M608425200 17085435

[23] Kumar S Sharma P Kumar D Chakraborty G Gorain M Kundu GC. Functional characterization of stromal osteopontin in melanoma progression and metastasis. PloS One. (2013) 8:e69116. doi: 10.1371/journal.pone.0069116 23935934 PMC3720680

[24] Klement JD Paschall AV Redd PS Ibrahim ML Lu C Yang D. An osteopontin/CD44 immune checkpoint controls CD8+ T cell activation and tumor immune evasion. J Clin Invest. (2018) 128:5549–60. doi: 10.1172/JCI123360 PMC6264631 30395540

[25] Ma C Komohara Y Ohnishi K Shimoji T Kuwahara N Sakumura Y. Infiltration of tumor-associated macrophages is involved in CD44 expression in clear cell renal cell carcinoma. Cancer Sci. (2016) 107:700–7. doi: 10.1111/cas.12917 PMC4970838 26918621

[26] Xiao Y Yang K Wang Z Zhao M Deng Y Ji W. CD44-mediated poor prognosis in glioma is associated with M2-polarization of tumor-associated macrophages and immunosuppression. Front Surg. (2021) 8:775194. doi: 10.3389/fsurg.2021.775194 35187044 PMC8850306

[27] Farajzadeh R Zarghami N Serati-Nouri H Momeni-Javid Z Farajzadeh T Jalilzadeh-Tabrizi S. Macrophage repolarization using CD44-targeting hyaluronic acid-polylactide nanoparticles containing curcumin. Artif cells nanomedicine Biotechnol. (2018) 46:2013–21. doi: 10.1080/21691401.2017.1408116 29183161

[28] Spaeth EL Labaff AM Toole BP Klopp A Andreeff M Marini FC. Mesenchymal CD44 expression contributes to the acquisition of an activated fibroblast phenotype *via* TWIST activation in the tumor microenvironment. Cancer Res. (2013) 73:5347–59. doi: 10.1158/0008-5472.CAN-13-0087 PMC3767181 23838935

[29] He C Sheng L Pan D Jiang S Ding L Ma X. Single-cell transcriptomic analysis revealed a critical role of SPP1/CD44-mediated crosstalk between macrophages and cancer cells in glioma. Front Cell Dev Biol. (2021) 9:779319. doi: 10.3389/fcell.2021.779319 34805184 PMC8602110

[30] Liu L Zhang R Deng J Dai X Zhu X Fu Q. Construction of TME and Identification of crosstalk between Malignant cells and macrophages by SPP1 in hepatocellular carcinoma. Cancer Immunol Immunother CII. (2022) 71:121–36. doi: 10.1007/s00262-021-02967-8 PMC10992184 34028567

[31] Xie W Cheng J Hong Z Cai W Zhuo H Hou J. Multi-Transcriptomic analysis reveals the heterogeneity and tumor-Promoting role of SPP1/CD44-Mediated intratumoral crosstalk in gastric cancer. Cancers. (2022) 15:164–92. doi: 10.3390/cancers15010164 PMC9818284 36612160

[32] Sillanpää S Anttila MA Voutilainen K Tammi RH Tammi MI Saarikoski SV. CD44 expression indicates favorable prognosis in epithelial ovarian cancer. Clin Cancer Res. (2003) 9:5318–24. 14614016

[33] Tjhay F Motohara T Tayama S Narantuya D Fujimoto K Guo J. CD44 variant 6 is correlated with peritoneal dissemination and poor prognosis in patients with advanced epithelial ovarian cancer. Cancer Sci. (2015) 106:1421–8. doi: 10.1111/cas.12765 PMC4638001 26250934

[34] Ahmed MA Aleskandarany MA Rakha EA Moustafa RZ Benhasouna A Nolan C. A CD44^-^/CD24^+^ phenotype is a poor prognostic marker in early invasive breast cancer. Breast Cancer Res Treat. (2012) 133:979–95. doi: 10.1007/s10549-011-1865-8 22119938

[35] Wang H Wang L Song Y Wang S Huang X Xuan Q. CD44(+)/CD24(-) phenotype predicts a poor prognosis in triple-negative breast cancer. Oncol Lett. (2017) 14:5890–8. doi: 10.3892/ol PMC5661458 29113223

[36] Roosta Y Sanaat Z Nikanfar AR Dolatkhah R Fakhrjou A. Predictive value of CD44 for prognosis in patients with breast cancer. Asian Pacific J Cancer Prev APJCP. (2020) 21:2561–7. doi: 10.31557/APJCP.2020.21.9.2561 PMC7779424 32986353

[37] Gu J Chen D Li Z Yang Y Ma Z Huang G. Prognosis assessment of CD44(+)/CD24(-) in breast cancer patients: a systematic review and meta-analysis. Arch gynecology obstetrics. (2022) 306:1147–60. doi: 10.1007/s00404-022-06402-w 35435483

[38] Wu G Song X Liu J Li S Gao W Qiu M. Expression of CD44 and the survival in glioma: a meta-analysis. Bioscience Rep. (2020) 40:520–30. doi: 10.1042/BSR20200520 PMC7160241 32232385

[39] Dong Q Li Q Wang M Hu J Dai J Niu L. Elevated CD44 expression predicts poor prognosis in patients with low-grade glioma. Oncol Lett. (2019) 18:3698–704. doi: 10.3892/ol PMC6732950 31516582

[40] Kobayashi K Matsumoto H Matsuyama H Fujii N Inoue R Yamamoto Y. Clinical significance of CD44 variant 9 expression as a prognostic indicator in bladder cancer. Oncol Rep. (2016) 36:2852–60. doi: 10.3892/or.2016.5061 27599396

[41] Ghaffarzadehgan K Jafarzadeh M Raziee HR Sima HR Esmaili-Shandiz E Hosseinnezhad H. Expression of cell adhesion molecule CD44 in gastric adenocarcinoma and its prognostic importance. World J Gastroenterol. (2008) 14:6376–81. doi: 10.3748/wjg.14.6376 PMC2766121 19009655

[42] Cao X Cao D Jin M Jia Z Kong F Ma H. CD44 but not CD24 expression is related to poor prognosis in non-cardia adenocarcinoma of the stomach. BMC Gastroenterol. (2014) 14:157. doi: 10.1186/1471-230X-14-157 25212506 PMC4175630

[43] Dubey P Gupta R Mishra A Kumar V Bhadauria S Bhatt MLB. Evaluation of correlation between CD44, radiotherapy response, and survival rate in patients with advanced stage of head and neck squamous cell carcinoma (HNSCC). Cancer Med. (2022) 11:1937–47. doi: 10.1002/cam4.4497 PMC9089225 35274800

[44] Li X Ma X Chen L Gu L Zhang Y Zhang F. Prognostic value of CD44 expression in renal cell carcinoma: a systematic review and meta-analysis. Sci Rep. (2015) 5:13157. doi: 10.1038/srep13157 26287771 PMC4541415

[45] Luo Y Tan Y. Prognostic value of CD44 expression in patients with hepatocellular carcinoma: meta-analysis. Cancer Cell Int. (2016) 16:47. doi: 10.1186/s12935-016-0325-2 27330410 PMC4912706

[46] Liu Y Wu T Lu D Zhen J Zhang L. CD44 overexpression related to lymph node metastasis and poor prognosis of pancreatic cancer. Int J Biol Markers. (2018) 33(3):308–13. doi: 10.1177/1724600817746951 29683068

[47] Cortes-Dericks L Schmid R. CD44 and its ligand hyaluronan as potential biomarkers in Malignant pleural mesothelioma: evidence and perspectives. Respir Res. (2017) 18(1):58. doi: 10.1186/s12931-017-0546-5 28403901 PMC5389171

[48] Lee S Harn H Lin T Yeh K Liu Y Tsai C. Prognostic significance of CD44v5 expression in human thymic epithelial neoplasms. Ann Thorac Surg. (2003) 76(1):213–218; discussion 218. doi: 10.1016/S0003-4975(03)00319-9 12842543

[49] Modi S Kulkarni V. Discovery of VEGFR-2 inhibitors exerting significant anticancer activity against CD44+ and CD133+ cancer stem cells (CSCs): Reversal of TGF-β induced epithelial-mesenchymal transition (EMT) in hepatocellular carcinoma. Eur J Med Chem. (2020) 207:112851. doi: 10.1016/j.ejmech.2020.112851 33002846

[50] Zhang H Brown R Wei Y Zhao P Liu S Liu X. CD44 splice isoform switching determines breast cancer stem cell state. Genes Dev. (2019) 33:166–79. doi: 10.1101/gad.319889.118 PMC6362815 30692202

[51] Gomez KE Wu F Keysar SB Morton JJ Miller B Chimed TS. Cancer cell CD44 mediates macrophage/Monocyte-Driven regulation of head and neck cancer stem cells. Cancer Res. (2020) 80:4185–98. doi: 10.1158/0008-5472.CAN-20-1079 PMC8146866 32816856

[52] Ivanova EL Costa B Eisemann T Lohr S Boskovic P Eichwald V. CD44 expressed by myeloid cells promotes glioma invasion. Front Oncol. (2022) 12:969787. doi: 10.3389/fonc.2022.969787 35992852 PMC9386454

[53] Witschen PM Chaffee TS Brady NJ Huggins DN Knutson TP LaRue RS. Tumor cell associated hyaluronan-CD44 signaling promotes pro-Tumor inflammation in breast cancer. Cancers. (2020) 12:1325–48. doi: 10.3390/cancers12051325 PMC7281239 32455980

[54] Zhang Q Liu Y Wang X Zhang C Hou M Liu Y. Integration of single-cell RNA sequencing and bulk RNA transcriptome sequencing reveals a heterogeneous immune landscape and pivotal cell subpopulations associated with colorectal cancer prognosis. Front Immunol. (2023) 14:1184167. doi: 10.3389/fimmu.2023.1184167 37675100 PMC10477986

[55] Jhunjhunwala S Hammer C Delamarre L. Antigen presentation in cancer: insights into tumour immunogenicity and immune evasion. Nat Rev Cancer. (2021) 21:298–312. doi: 10.1038/s41568-021-00339-z 33750922

[56] Morad G Helmink BA Sharma P Wargo JA. Hallmarks of response, resistance, and toxicity to immune checkpoint blockade. Cell. (2022) 185:576. doi: 10.1016/j.cell.2022.01.008 35120665

[57] Ma W Pham B Li T. Cancer neoantigens as potential targets for immunotherapy. Clin Exp metastasis. (2022) 39:51–60. doi: 10.1007/s10585-021-10091-1 33950415 PMC8097110

[58] Gjoerup O Brown CA Ross JS Huang RSP Schrock A Creeden J. Identification and utilization of biomarkers to predict response to immune checkpoint inhibitors. AAPS J. (2020) 22:132. doi: 10.1208/s12248-020-00514-4 33057937

