# Supplementary material for: Pan-cancer and single-cell analyses identify CD44 as an immunotherapy response predictor and regulating macrophage polarization and tumor progression in colorectal cancer
Source: Front Oncol. 2024 Mar 25;14:1380821. doi: 10.3389/fonc.2024.1380821 (PMC10999581; doi:10.3389/fonc.2024.1380821)
Supplement: Supplementary file 1 [file DataSheet_1.docx]

Supplementary Material

**Abbreviations:**

ACC, Adrenocortical carcinoma

BLCA, Bladder urothelial carcinoma

BRCA, Breast invasive carcinoma

CD44, Cluster of differentiation 44

CHOL, Cholangiocarcinoma

COAD, Colon adenocarcinoma

CRC, Colorectal cancer

DSS, Disease-specific survival

ESCA, Esophageal carcinoma

GBM, Glioblastomamultiforme

GEO, Gene Expression Omnibus

HPA, Human Protein Atlas

HSNC, Head and neck squamous cell carcinoma

ICB, Immune checkpoint blockade

ICI, Immune checkpoint inhibitor

KICH, Kidney chromophobe

KIRC, Kidney renal clear cell carcinoma

KIRP, Kidney renal papillary cell carcinoma

LIHC, Hepatocellular carcinoma

LUAD, Lung adenocarcinoma

MSI, Microsatellite instability

NSCLC, Non-small cell lung cancer

OPN, Osteopontin

OS, Overall survival

OV, Ovarian serous cystadenocarcinoma

PAAD, Pancreatic adenocarcinoma

PIPG, Pheochromocytoma and paraganglioma

PRAD, Prostate adenocarcinoma

READ, Rectum adenocarcinoma

SKCM, Skin cutaneous melanoma

STAD, Stomach adenocarcinoma

TCGA, The Cancer Genome Atlas

THCA, Thyroid carcinoma

THYM, Thymic epithelial neoplasms

TIDE, Tumor immune dysfunction and exclusion

TMB, Tumor mutational burden

TME, Tumor microenvironment

UCEC, Uterine corpus endometrial carcinoma

[Figure S1.](https://static-content.springer.com/esm/art%3A10.1186%2Fs12935-022-02483-4/MediaObjects/12935_2022_2483_MOESM1_ESM.pdf) Flow chart of the research.


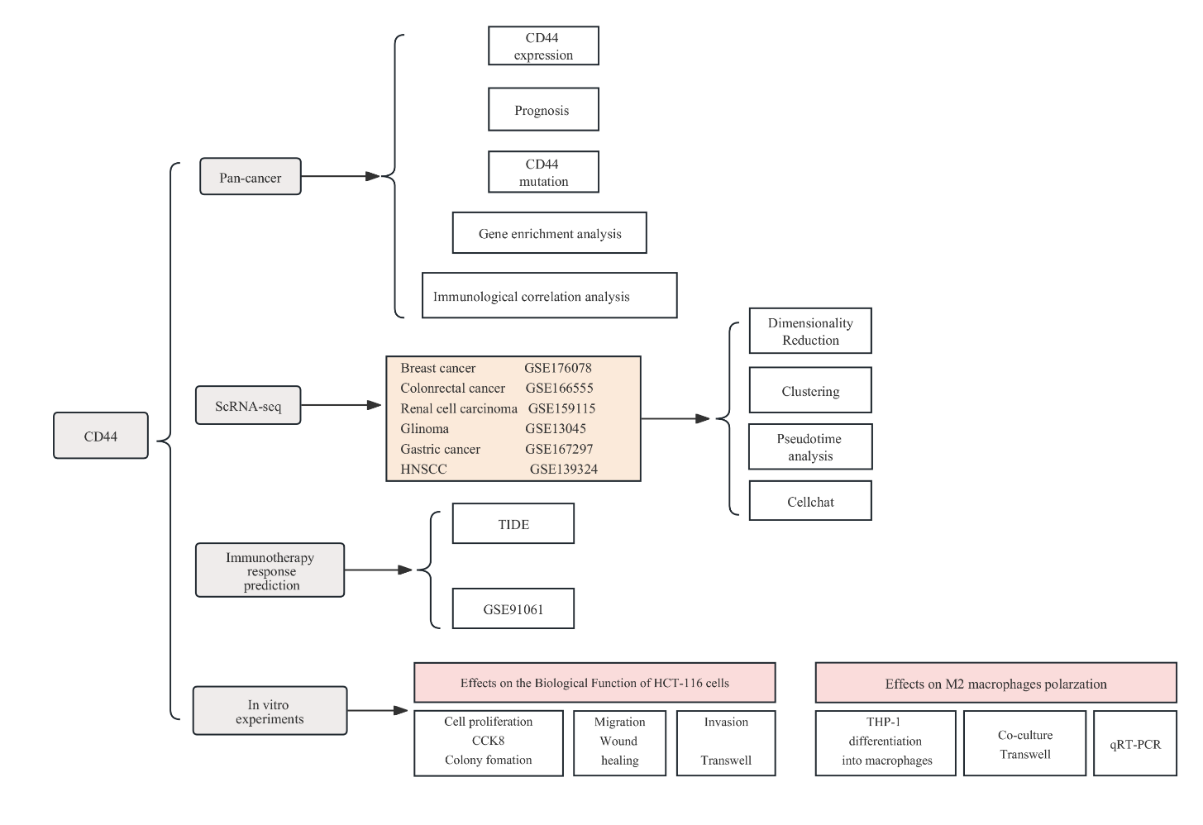


[Table S1.](https://static-content.springer.com/esm/art%3A10.1186%2Fs12935-022-02483-4/MediaObjects/12935_2022_2483_MOESM1_ESM.pdf) siRNA sequence and PCR primer sequence.

| siRNA sequence and PCR primer sequence | | |
| --- | --- | --- |
| siRNA | sense (5'-3') | antisense(5'-3') |
| siRNA1 | CUCCCAGUAUGACACAUAU(dT)(dT) | AUAUGUGUCAUACUGGGAG(dT)(dT) |
| siRNA2 | GGACCAAUUACCAUAACUA(dT)(dT) | UAGUUAUGGUAAUUGGUCC(dT)(dT) |
| primer | forward(5'-3') | reverse(5'-3') |
| CD44 | TGACAACGCAGCAGAGTAATTC | TTCCACCTGTGACATCATTCCT |
| CD86 | CCCCAGACCACATTCCTTGG | TGTTCACTCTCTTCCCTCTCCA |
| CD163 | CCAGTCCCAAACACTGTCCT | CACTCTCTATGCAGGCCACA |
| CD206 | TACTGAACCCCCACAACTGC | ACCAGAGAGGAACCCATTCG |
| IL-6 | TAGTCCTTCCTACCCCAATTTC | TTGGTCCTTAGCCACTCCTTC |
| IL-10 | TTCTTTCAAACAAAGGACCAGC | GCAACCCAAGTAACCCTTAAAG |
| TNFα | CTCCCAGGTCCTCTTCAAGG | TTGATGGCAGAGAGGAGGTT |
| TGFβ | CCCTGGACACCAACTATTGC | GCAGAAGTTGGCATGGTAGC |
| GAPDH | GTCTCCTCTGACTTCAACAGCG | ACCACCCTGTTGCTGTAGCCAA |

[Table S2.](https://static-content.springer.com/esm/art%3A10.1186%2Fs12935-022-02483-4/MediaObjects/12935_2022_2483_MOESM1_ESM.pdf) **List of primary antibodies for western blot**

| Antibody | Company |
| --- | --- |
| CD44 | Abcam (ab189524) |
| CD86 | CST (91882) |
| CD206 | CST (24595) |
| TNF α | Wanleibio (WL01518) |
| TGF β | MCE (HY-P80521) |
| GAPDH | Abcam (ab181602) |
|  |  |


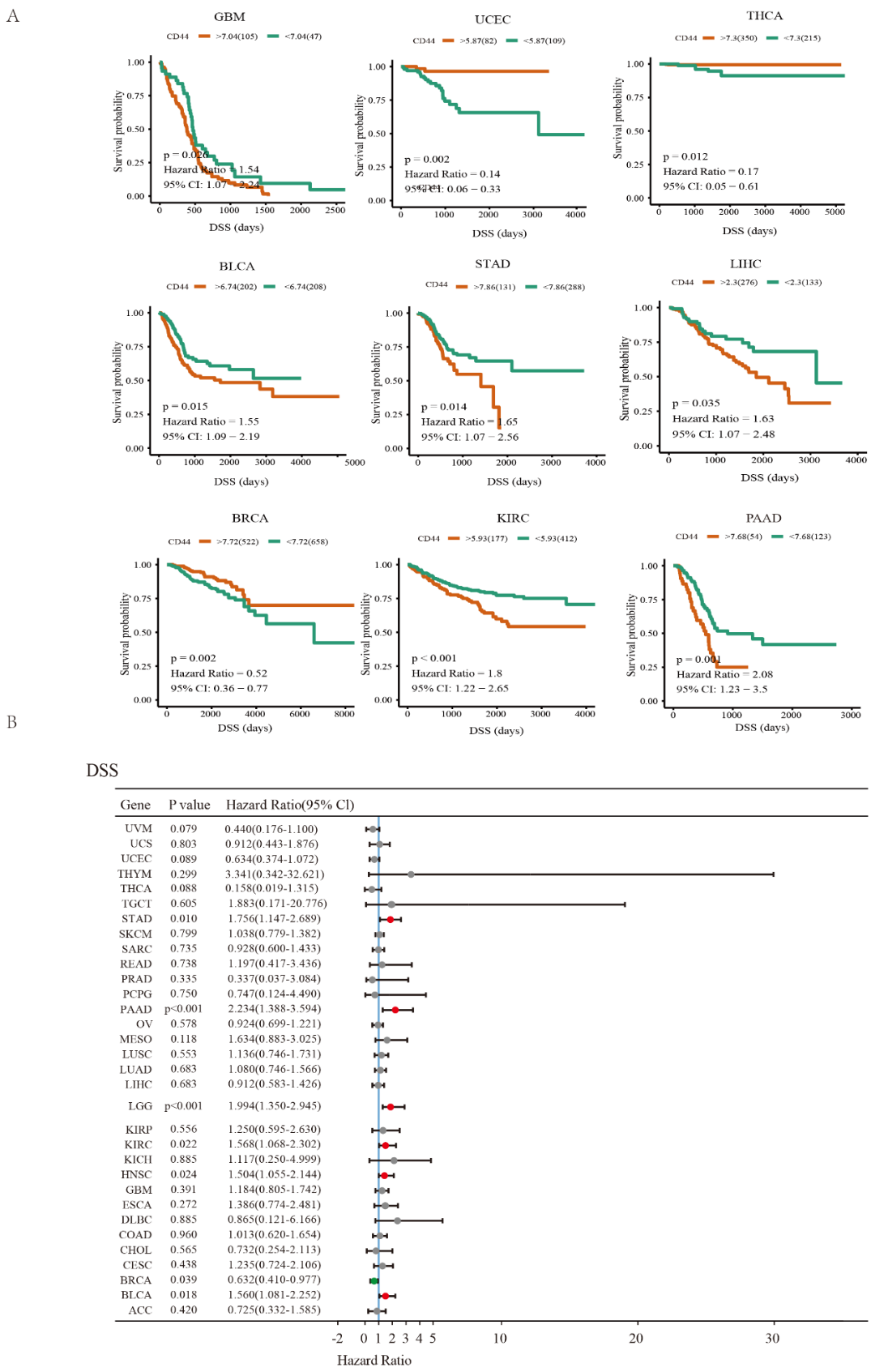


[Figure S2.](https://static-content.springer.com/esm/art%3A10.1186%2Fs12935-022-02483-4/MediaObjects/12935_2022_2483_MOESM1_ESM.pdf) The relationship between the expression of CD44 and the DSS prognosis value in pan-cancer. (A). Kaplan-Meier survival curves of the CD44 expression associated with DSS in different cancers.(B). Forest plots of univariate Cox regression analyses of the prognostic role of CD44 in pan-cancer DSS.


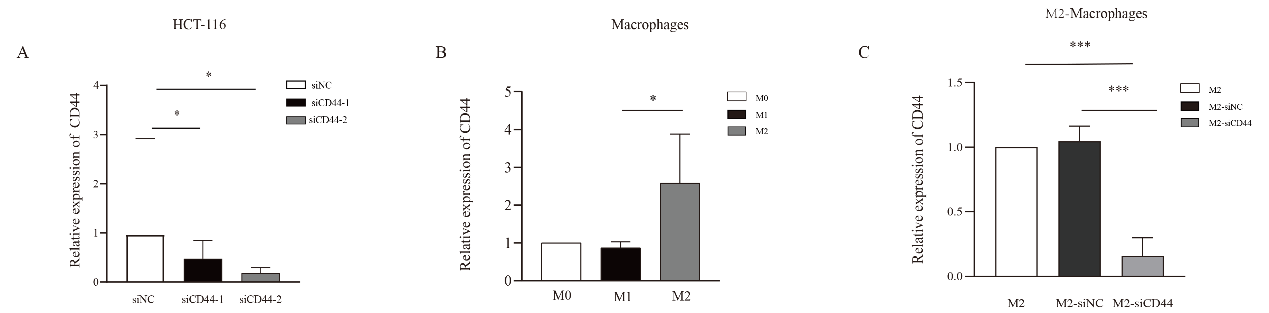


[Figure S3.](https://static-content.springer.com/esm/art%3A10.1186%2Fs12935-022-02483-4/MediaObjects/12935_2022_2483_MOESM1_ESM.pdf) Relative mRNA expression level of CD44 in HCT-116 cells and Macrophages.(A). Relative expression of CD44 in HCT-116 cells transfected with NC or CD44 siRNA.(B). Relative expression of CD44 in M0, M1 and M2 macrophages. (C). Relative expression of CD44 in M2 macrophages transfected with NC or CD44 siRNA.The experiments were performed in triplicate, and the data are expressed by mean ± SD , using one-way ANOVA , *P < 0.05, * *P < 0.01, * **P < 0.001.


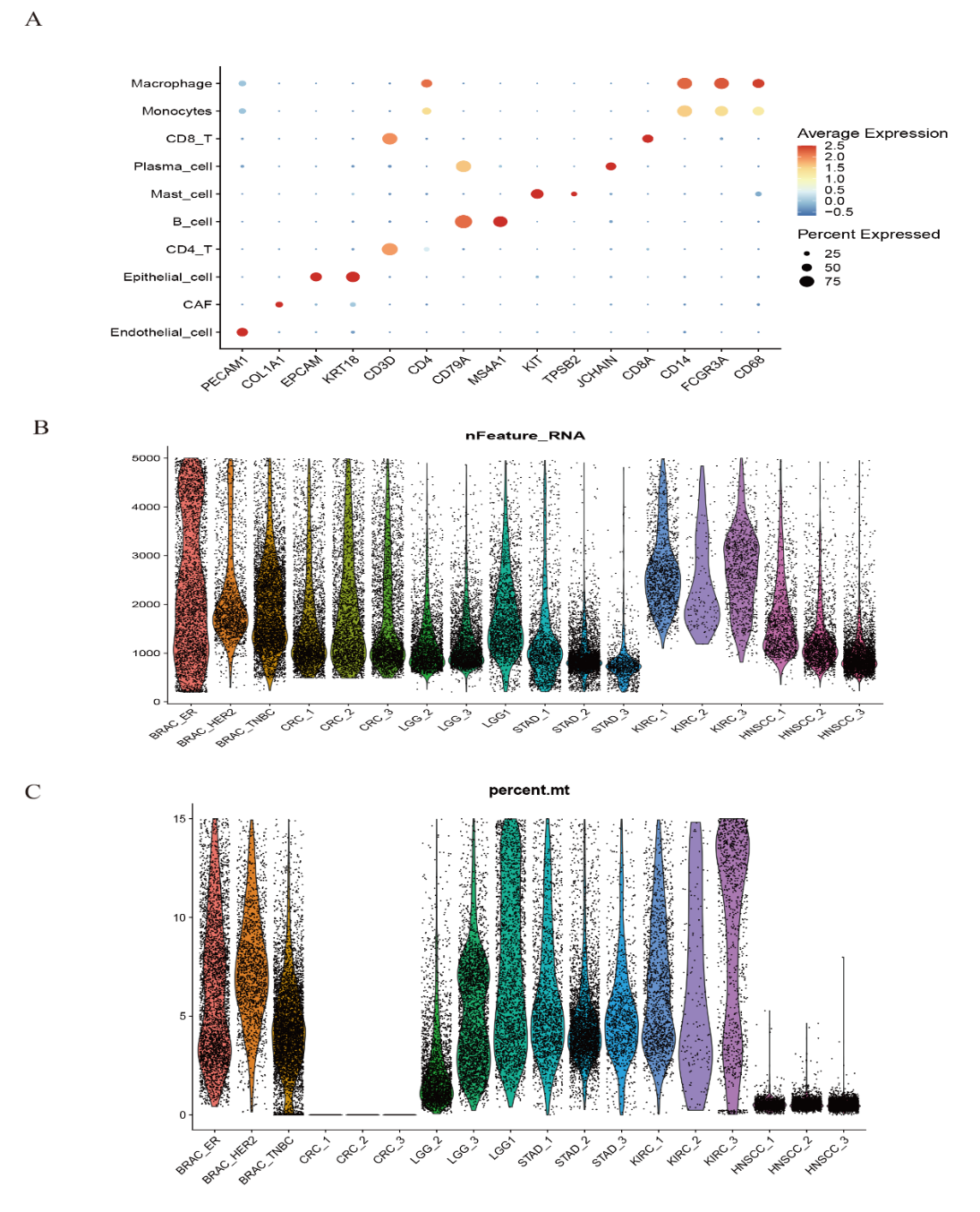


Figure S4. ScRNA-seq data analysis annotation chart and quality control chart.

(A) Bubble plot of marker genes for cell clusters annotation. The color of the dot represents the marker genes expression, and the size of the dot represents to p-value.

(B) Violin plot of the distribution of cells with gene >200 and gene < 5000.

(C) Violin plot of the percentage of mitochondrial gene in cells.


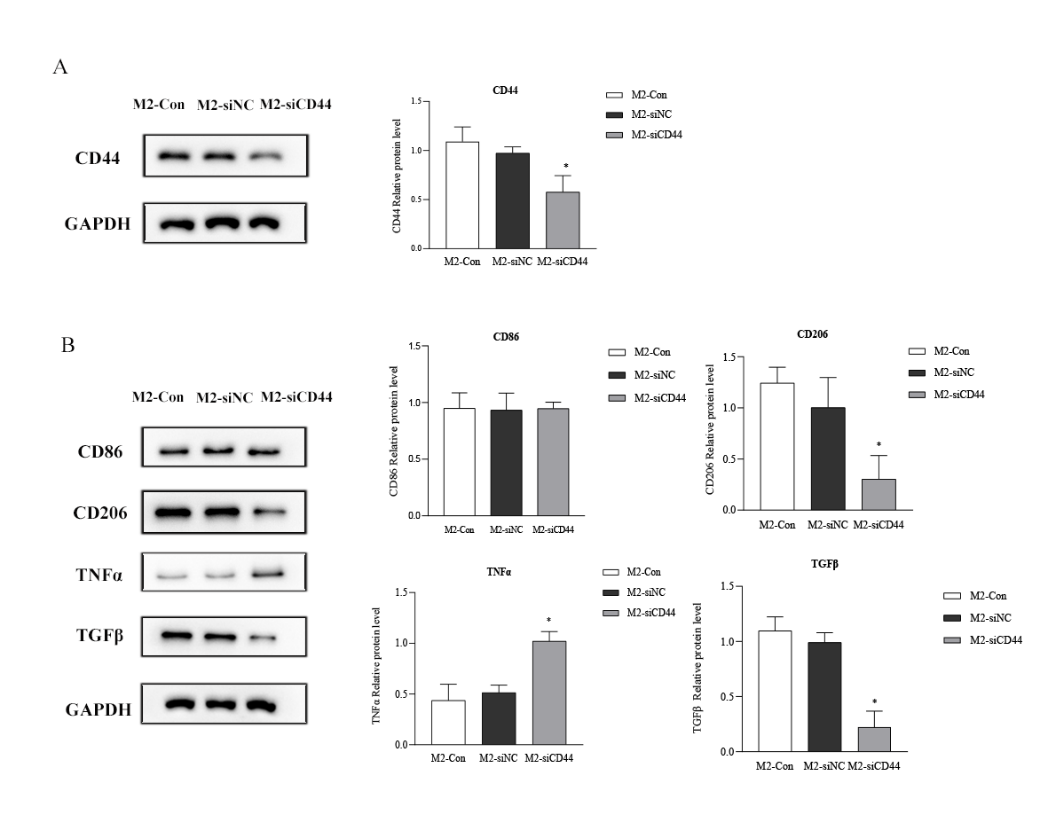


Figure S5.The effect of siRNA transfection on the expression of target proteins in M2 macrophages were analyzed by Western blot.

(A) Compared with M2 macrophages in the control group, siNC transfection did not affect the expression of target proteins. siCD44 transfection significantly decreased the expression of CD44 protein in M2 macrophages.

(B) Knockdown of CD44 in M2 macrophages was accompanied by decreased the protein expression of M2 markers CD206 and TGFβ. Meanwhile, the protein expression of M1 marker CD86 did not show significant changes, whereas TNF α The expression level of significantly increased.

The representative data are presented as the mean±SD, *P < 0.05, **P < 0.01, ***P < 0.001.
